# The phosphoinositide 3-kinase inhibitor alpelisib restores actin organization and improves proximal tubule dysfunction *in vitro* and in a mouse model of Lowe syndrome and Dent disease

**DOI:** 10.1016/j.kint.2020.05.040

**Published:** 2020-10

**Authors:** Marine Berquez, Jonathan R. Gadsby, Beatrice Paola Festa, Richard Butler, Stephen P. Jackson, Valeria Berno, Alessandro Luciani, Olivier Devuyst, Jennifer L. Gallop

**Affiliations:** 1 Institute of Physiology, University of Zurich, Zurich, Switzerland; 2 Gurdon Institute and Department of Biochemistry, University of Cambridge, Cambridge, UK; 3 Gurdon Institute, University of Cambridge, Cambridge, UK; 4 Experimental Imaging Center, ALEMBIC, IRCCS San Raffaele Scientific Institute, Milan, Italy

**Keywords:** cytoskeleton, endocytosis, lipids, proximal tubule, renal Fanconi syndrome

## Abstract

Loss-of-function mutations in the *OCRL* gene, which encodes the phosphatidylinositol [PI] 4,5-bisphosphate [PI(4,5)P_2_] 5-phosphatase OCRL, cause defective endocytosis and proximal tubule dysfunction in Lowe syndrome and Dent disease 2. The defect is due to increased levels of PI(4,5)P_2_ and aberrant actin polymerization, blocking endosomal trafficking. PI 3-phosphate [PI(3)P] has been recently identified as a coactivator with PI(4,5)P_2_ in the actin pathway. Here, we tested the hypothesis that phosphoinositide 3-kinase (PI3K) inhibitors may rescue the endocytic defect imparted by OCRL loss, by rebalancing phosphoinositide signals to the actin machinery. The broad-range PI3K inhibitor copanlisib and class IA p110α PI3K inhibitor alpelisib reduced aberrant actin polymerization in OCRL-deficient human kidney cells *in vitro*. Levels of PI 3,4,5-trisphosphate, PI(4,5)P_2_ and PI(3)P were all reduced with alpelisib treatment, and siRNA knockdown of the PI3K catalytic subunit p110α phenocopied the actin phenotype. In a humanized *Ocrl*^*Y/-*^ mouse model, alpelisib reduced endosomal actin staining while restoring stress fiber architecture and levels of megalin at the plasma membrane of proximal tubule cells, reflected by improved endocytic uptake of low molecular weight proteins *in vivo*. Thus, our findings support the link between phosphoinositide lipids, actin polymerization and endocytic trafficking in the proximal tubule and represent a proof-of-concept for repurposing alpelisib in Lowe syndrome/Dent disease 2.

## Graphical abstract

**Figure:**
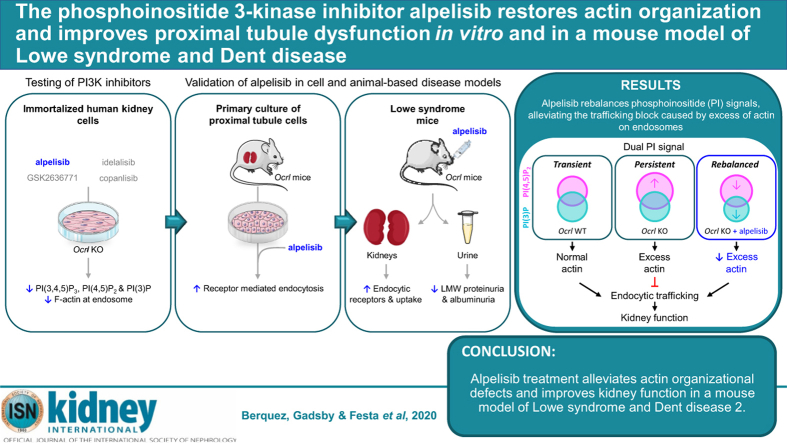

see commentary on page 824

**Translational Statement**

Lowe syndrome and Dent disease 2, caused by mutations in the phosphoinositide lipid 5-phosphatase OCRL, manifest with proximal tubule dysfunction and low-molecular-weight proteinuria with only supportive care available. Our findings reveal that the phosphoinositide 3-kinase inhibitor alpelisib, which is currently approved for cancer therapy, alleviates the aberrant phosphoinositide balance and actin phenotype associated with the OCRL loss, causing a substantial improvement of the endocytic machinery and absorptive capacity in cellular systems and a humanized mouse model for Lowe syndrome/Dent disease 2. Given its apparent safety profile, alpelisib is a promising candidate for drug repurposing in Lowe syndrome and Dent disease.

Epithelial cells lining the proximal tubules (PTs) of the kidney possess an efficient receptor-mediated endolysosomal pathway that recovers and processes essential substances that are filtered through the glomerulus. Congenital disorders affecting endolysosomes cause PT dysfunction (renal Fanconi syndrome) with urinary loss of solutes and low-molecular-weight (LMW) proteins, often complicated by metabolic and growth complications and development of chronic kidney disease.^1^ Inactivating mutations in *OCRL* have been associated with Dent disease 2 (MIM #300555), a disorder characterized by PT dysfunction, kidney stones, and progressive kidney failure, and with the oculocerebrorenal syndrome of Lowe (MIM #309000), which displays, in addition to the PT dysfunction and kidney failure, systemic manifestations such as congenital cataracts, cognitive disability, and hypotonia.^2^, ^3^, ^4^ Current treatments for Dent disease 2 and Lowe syndrome are only supportive.

LMW proteinuria is a consistent feature observed in Lowe syndrome/Dent disease 2, revealing that *OCRL* affects receptor-mediated endocytosis.^5^ *OCRL* encodes the phosphatidylinositol (PI) 4,5-bisphosphate [PI(4,5)P_2_] 5-phosphatase OCRL,^6^ which controls lipid identity in the endolysosomal pathway by degrading PI(4,5)P_2_ (Figure 1a). Deficient degradation of PI(4,5)P_2_ through OCRL is implicated in the failure to uncoat clathrin-coated vesicles in fibroblasts, accompanied by the formation of “comet” structures of polymerized, filamentous actin from the resulting aberrant early endosome-like organelles.^7^, ^8^, ^9^ In other cell types, most notably the PT cells of the kidney, OCRL deficiency results in F-actin “basket” structures surrounding aberrant endolysosomal organelles.^10^ The excess F-actin blocks membrane trafficking through the endocytic and endolysosomal pathway, and is suggested to decrease recycling of the multiligand receptor megalin to the apical membrane, aggravating the defect within the endolysosomal pathway.^11^ In agreement with a prime role of aberrant F-actin, defective endocytic uptake caused by *OCRL* mutations is alleviated by targeting the actin machinery with latrunculin B, or by depleting actin regulatory proteins as well as by small, interfering RNA (siRNA)-mediated depletion of PI(4)P 5-kinase to adjust the synthesis of PI(4,5)P_2_^7^^,^^10^ (Figure 1a).

**Figure 1:**
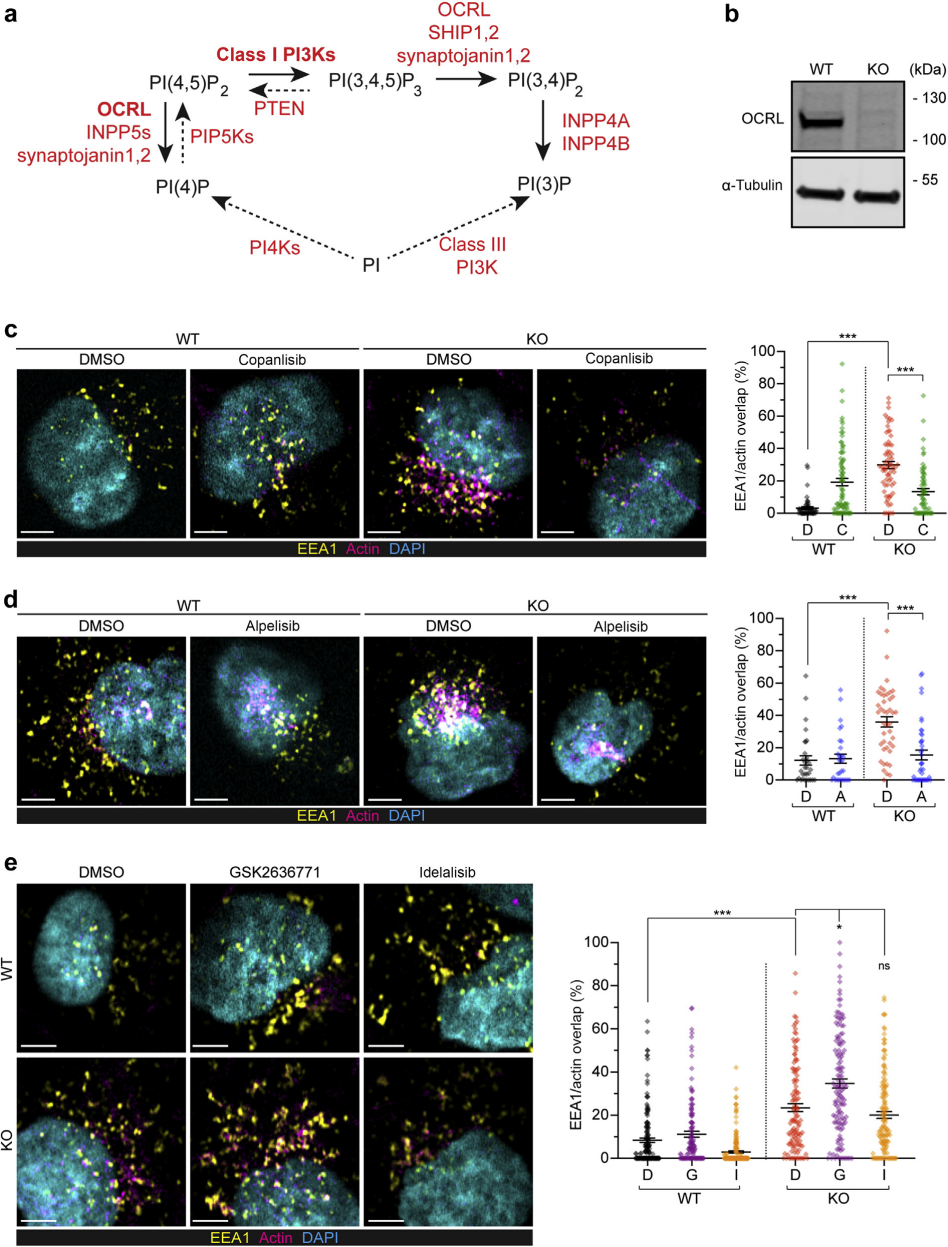
**PI3K inhibitors relieve aberrant actin assembly at endosomes in an OCRL-deficient human kidney (HK2) cell model.** (**a**) Steps of phosphoinositide lipid conversion relevant for this study indicating the conversions between PI(4,5)P_2_, PI(3,4,5)P_2_, and PI(3)P with the most relevant enzymes in bold, most relevant conversions in solid lines, and others in dashed lines. PI(4,5)P_2_ is elevated in Lowe syndrome due to the lack of 5-phosphatase activity from OCRL and is made from the phosphorylation of PI by phosphatidylinositol 4-kinases (PI4Ks) and PI(4)P 5-kinase (PIP5K). PI(4,5)P_2_ is phosphorylated by class I PI3Ks to produce PI(3,4,5)P_3_. PI(3,4,5)P_3_ can be dephosphorylated to PI(4,5)P_2_ by PTEN or to PI(3,4)P_2_ by SH-2–containing inositol 5′ polyphosphatase (SHIP) 1 and 2, synaptojanin 1 and 2, and also OCRL, although this is thought to be minor. PI(3,4)P_2_ is dephosphorylated to PI(3)P by inositol polyphosphate-4-phosphatase type I A (INPP4A) and B. PI(3)P is also made at endosomes via the phosphorylation of PI by class III PI3K, vacuolar sorting protein Vps34. (**b**) Western blots illustrating OCRL expression loss in the HK2 *OCRL* CRISPR knockout (KO) cell line compared with wild-type (WT) HK2 control cells with α-tubulin as loading control. (**c–e**) Representative Airyscan confocal micrographs and quantification of early endosome antigen 1 (EEA1)/actin overlap (expressed as a percentage of total detected EEA1+ vesicles) for HK2 WT or *OCRL* KO cells treated with either dimethylsulfoxide (DMSO) (**d**) or the indicated inhibitor and fixed using the 4% formaldehyde fix. In all cases, the images illustrate a single z-slice from an Airyscan-processed confocal stack of cells immunolabeled for EEA1 (yellow), phalloidin (actin, magenta), and 4′,6-diamidino-2-phenylindole (DAPI) (cyan). Bars = 5 μm. In the quantifications, the lines indicate the mean ± SEM and each data point is an individual cell. In all experiments, treatments were applied 16 hours before fixation. In all quantifications, statistical significance was assessed by a Kruskal-Wallis (K-W) analysis of variance with Dunn’s multiple comparisons test. (**c**) WT or KO cells treated with either DMSO or 100 nM of copanlisib, demonstrating rescue of the actin-endosomal overlap. K-W test: ∗∗∗*P* < 0.001, multiple comparisons; WT DMSO versus KO DMSO, KO DMSO versus KO copanlisib both ∗∗∗*P* < 0.001, WT copanlisib versus KO copanlisib *P* = 0.44 (not significant [ns]). N = 52, 85, 67, and 63 cells for WT DMSO, WT copanlisib, KO DMSO, and KO copanlisib, respectively. (**d**) WT or KO cells treated with either DMSO or 10 μM of alpelisib, demonstrating rescue of the actin-endosomal overlap. K-W test: ∗∗∗*P* < 0.001, multiple comparisons; WT DMSO versus KO DMSO, KO DMSO versus KO alpelisib both ∗∗∗*P* < 0.001, WT alpelisib versus KO alpelisib. *P* > 0.99 (ns). N = 31, 30, 43, and 41 cells for WT DMSO, WT alpelisib, KO DMSO, and KO alpelisib, respectively. (**e**) WT or KO cells treated with either DMSO, 10 μM of GSK2636771, or 10 μM of idelalisib, demonstrating that neither compound is able to significantly reduce the actin-endosomal overlap. K-W test: ∗∗∗*P* < 0.001, multiple comparisons; WT DMSO versus KO DMSO, ∗∗∗*P* < 0.001, KO DMSO versus KO GSK, ∗*P* = 0.04, KO DMSO versus KO idelalisib, *P* > 0.99 (ns). N = 159, 130, 203, 122, 131, and 145 cells for WT DMSO, WT GSK2636771, WT idelalisib, KO DMSO, KO GSK2636771, and KO idelalisib, respectively. To optimize viewing of this image, please see the online version of this article at www.kidney-international.org.

Actin polymerization is increasingly understood as arising not only from PI(4,5)P_2_ but also from other phosphoinositide lipids. For example, PI(3,4,5)P_3_ activates Rho-type GTPase Rac and PI(3)P acts in conjunction with PI(4,5)P_2_ to stimulate actin polymerization downstream of another Rho-type GTPase, Cdc42.^12^, ^13^, ^14^ The interconversion of these phosphoinositides is coupled to the movement of lipids through the endocytic and endosomal pathway. PI(4,5)P_2_ is phosphorylated by class I phosphatidylinositol-3′-kinase (PI3K) to PI(3,4,5)P_3_ at the plasma membrane and becomes progressively dephosphorylated to PI(3,4)P_2_ and PI(3)P or PI(4,5)P_2_.^14^^,^^15^ PI(3)P is enriched at endosomes, where it is essential for their function and predominantly made by direct by phosphorylation of PI by class III PI3K, Vps34^16^ (Figure 1a). In an OCRL-deficient retinal pigmented epithelial (RPE) cell line, actin comets generated are reduced by treatment with PI3K inhibitors wortmannin and Vps34-IN1, in agreement with biochemical studies showing a coregulation of actin via the coincidence of both PI(3)P and PI(4,5)P_2_.^14^

The rescue of endocytosis observed when targeting the actin machinery in OCRL patient and knockout (KO) cells,^7^^,^^10^ together with upstream regulation of actin by PI3K activity^14^ and the highly interconnected nature of PI conversion, provides an intriguing possibility that class I PI3K inhibitors may be of utility in Lowe syndrome. Such inhibitors, developed for cancer therapy, are approved for clinical use^17^ and are thus potentially amenable for drug repurposing. Although compounds such as copanlisib^18^ have a broad specificity and side effects, recent success has been demonstrated for more specific inhibitors. Idelalisib,^19^ which targets PI3K subunit p110δ, is approved for chronic lymphocytic lymphoma, and alpelisib, which targets p110α, is approved for use in breast cancer.^20^^,^^21^ Moreover, alpelisib has shown clinical benefit in children with PROS/CLOVES syndrome,^22^ a rare overgrowth syndrome resulting from activation of PIK3A.

Here, we tested the hypothesis that class I PI3K inhibitors may rescue the endocytic defect due to the loss of OCRL, using established cellular and mouse models.^10^^,^^11^^,^^23^ We show that the inhibition of PI3K activity via copanlisib or alpelisib, or siRNA-mediated depletion of the alpelisib-target catalytic subunit p110α reduces the excess actin polymerization in human kidney (HK2) cells deficient in OCRL. Focusing on alpelisib as the most specific compound for clinical use, we show that it reduces actin polymerization and improves uptake through the endolysosomal pathway in PT cells from humanized *Ocrl*^*Y/−*^ mice *in vitro*. Furthermore, alpelisib treatment *in vivo* alleviates proteinuria, reduces PT dysfunction, and rescues the cellular levels of megalin in humanized *Ocrl*^*Y/−*^ mice. These results support alpelisib as a candidate for drug repurposing in Lowe syndrome and Dent disease 2.

## Results

### Class I PI3K inhibitors reduce actin aggregation in OCRL-KO HK2 cells

We used CRISPR-Cas9 gene editing to knockout (KO) *OCRL* in the HK2 cell line to allow testing of class I PI3K inhibitors on the characteristic actin aggregation at endolysosomal compartments.^7^^,^^9^^,^^10^^,^^14^ Western blotting verified a 98.0 ± 0.7% reduction in OCRL expression in lysates from the KO HK2 cells (Figure 1b). The *OCRL* KO recapitulated the actin basket phenotype in the HK2 cells, as indicated by an increased overlap between F-actin and early endosome antigen 1 (EEA1), quantified on Airyscan confocal microscopy z-stacks (Figure 1c–e). This increased overlap observed in *OCRL* KO cells was reversed by treatment with copanlisib (C), a broad-range PI3K inhibitor that targets p110α and δ and to a lesser extent the p110β and γ isoforms (Figure 1c). By western blotting of HK2 whole cell extracts, we found that the PI3K p110 regulatory subunits α, β, and δ were all well expressed in both wild-type (WT) and KO cells, with only trace levels of the p110γ isoform present (Supplementary Figure S1A).

We distinguished the PI3K specificity of the inhibition of F-actin using alpelisib (A), a selective inhibitor of p110α, and GSK2636771 (G) and idelalisib (I), selective inhibitors of p110β and p110δ, respectively (Supplementary Figure S1A). Although alpelisib treatment gave a similar reduction in endosomal actin polymerization compared with copanlisib (Figure 1d), GSK2636771 and idelalisib had a little effect on actin accumulation (Figure 1e). The class I PI3K inhibitors had no increased toxicity on *OCRL* KO relative to unmodified cells, based on the MTT assay (Supplementary Figure S1B).

### Alpelisib and p110α knockdown rescue the actin phenotype in HK2 cells

**Figure 2:**
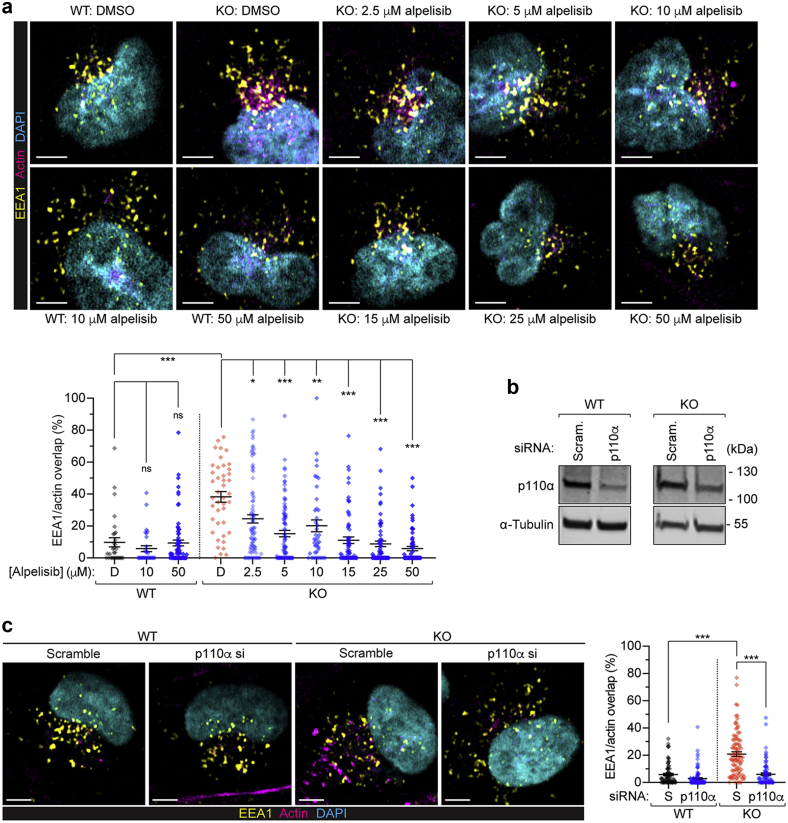
**Alpelisib effects on actin are dose-responsive and recapitulated by siRNA of PI3K p110α.** (**a**) Representative Airyscan confocal micrographs (fixed using the 4% formaldehyde fix and immunolabeled for early endosome antigen 1 [EEA1], yellow; actin [phalloidin], magenta; and 4′,6-diamidino-2-phenylindole [DAPI], cyan; bars = 5 μm) and quantification of wild-type (WT) or knockout (KO) human kidney (HK2) cells treated with dimethylsulfoxide (DMSO) or the indicated doses of alpelisib for 16 hours, demonstrating dose-responsive rescue of the actin-endosomal overlap. In all cases, the lines indicate mean ± SEM and the points indicate individual cells. N = 31, 30, 71, 42, 90, 89, 41, 69, 66, and 67 cells for WT control, WT 10 μM, WT 50 μM, KO DMSO, and KO 2.5, 5, 10, 15, 25, and 50 μM alpelisib, respectively. Statistical significance assessed by the Kruskal-Wallis (K-W) test with Dunn’s multiple comparisons test: overall ∗∗∗*P* < 0.001, multiple comparisons; WT DMSO versus KO DMSO, KO DMSO versus KO 5, 15, 25, and 50 μM alpelisib all ∗∗∗*P* < 0.001, KO DMSO versus KO 2.5 μM alpelisib ∗*P* = 0.0131, KO DMSO versus KO 10 μM alpelisib ∗∗*P* = 0.0043, WT DMSO versus both WT 10 and 50 μM alpelisib *P* > 0.9999 (not significant [ns]). (**b**) Western blot for p110α and the loading control α-tubulin of WT or KO cells treated with either scramble (Scram.) or p110α siRNA. (**c**) Representative Airyscan confocal micrographs (fixed using the 4% formaldehyde fix and immunolabeled for EEA1, yellow; actin [phalloidin], magenta; and DAPI, cyan; bars = 5 μm) and quantification of WT or KO cells treated with either scramble (S) or p110α siRNA, demonstrating reduction of EEA1-actin overlap on p110α depletion. K-W test with Dunn’s multiple comparisons test: ∗∗∗*P* < 0.001, multiple comparisons; WT scramble versus KO scramble, KO scramble versus KO p110α siRNA both ∗∗∗*P* < 0.001. N = 79, 108, 93, and 131 cells, respectively. HK2, human kidney; PI3K, phosphatidylinositol-3′-kinase; siRNA, small, interfering RNA. To optimize viewing of this image, please see the online version of this article at www.kidney-international.org.

For our ensuing studies, we focused on alpelisib as it is the most selective and least toxic PI3K inhibitor developed so far, and is currently used to treat a mosaic overactivation of PI3K class Iα in children with PROS/CLOVES syndrome.^22^ Alpelisib reduced the actin baskets in *OCRL* KO HK2 cells in a dose-responsive fashion (Figure 2a). The effects were observed at 10 μM dosage as soon as 4 hours after treatment, without apparent toxicity (Supplementary Figure S2). To test whether PI3K inhibition by alpelisib was the source of the decreased actin staining, we specifically reduced its target, the p110α subunit, using siRNA (Figure 2b). Compared with treatment with a control (sequence-scrambled) siRNA, siRNA against p110α yielded a 78.3 ± 4.8% and 68.9 ± 7.7% depletion of p110α protein in WT and *OCRL* KO HK2 cells, respectively, reflected by a significant reduction in actin baskets (Figure 2c).

### Reduced levels of PI(3,4,5)P_3_, PI(4,5)P_2_, and PI(3)P in alpelisib-treated HK2 cells

**Figure 3:**
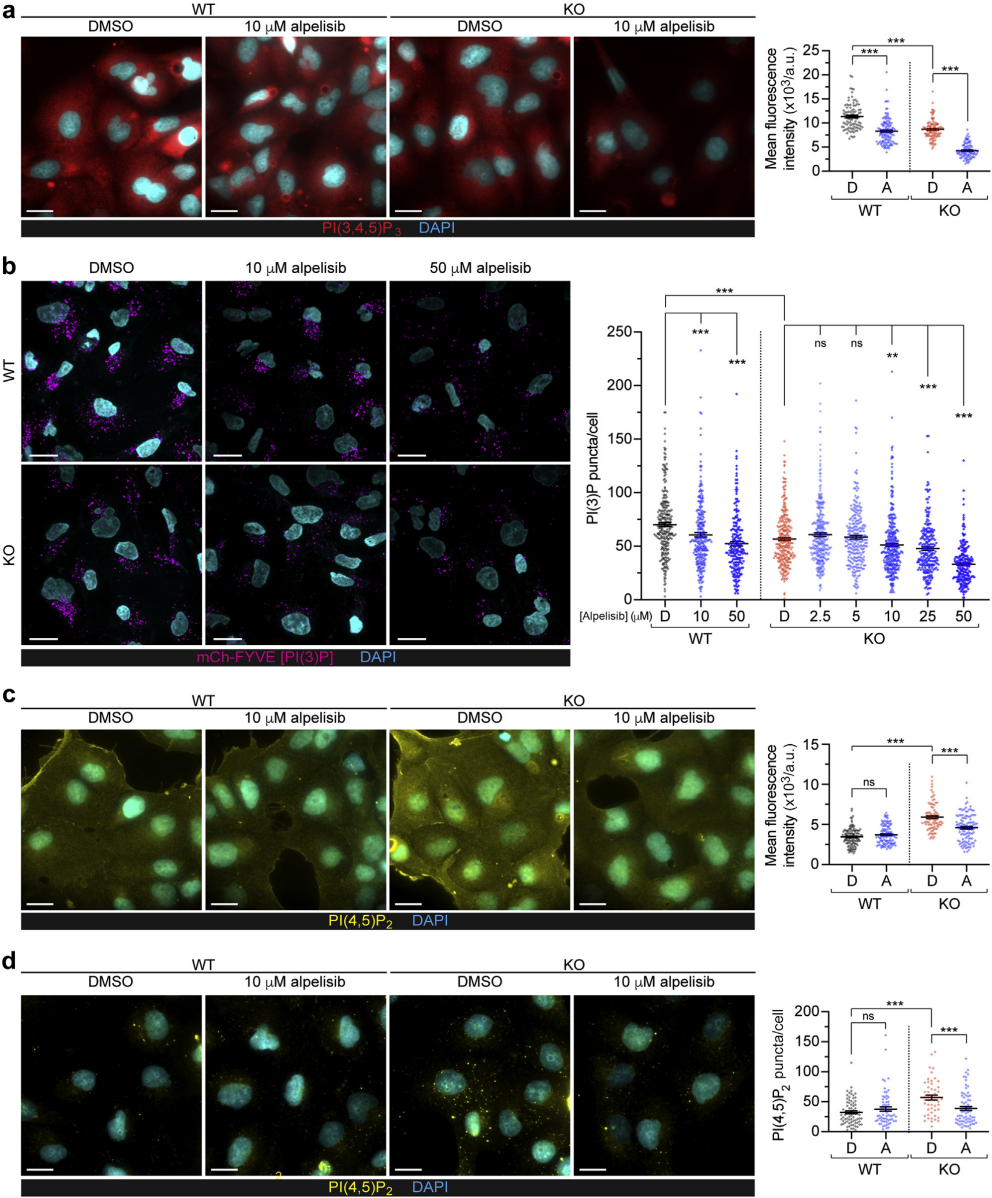
**Levels of phosphatidylinositol (PI) 4,5-bisphosphate [PI(4,5)P**_**2**_**] are elevated with OCRL knockout (KO), and PI(3,4,5)P**_**3**_**, PI(4,5)P**_**2**_**, and PI(3)P are suppressed by alpelisib treatment.** In all experiments, the indicated treatments were applied 16 hours before fixation. In the quantifications, the lines indicate the mean ± SEM and each data point results from an individual cell. In all quantifications, statistical significance was assessed by a Kruskal-Wallis (K-W) analysis of variance with Dunn’s multiple comparisons test. Bars = 20 μm in all images. (**a**) Representative widefield micrographs of wild-type (WT) or KO human kidney (HK2) cells treated with dimethylsulfoxide (DMSO) or 10 μM of alpelisib and then fixed with the plasma membrane fix and immunolabeled for PI(3,4,5)P_3_ (red) and 4′,6-diamidino-2-phenylindole (DAPI) (cyan), with quantification of the mean cellular PI(3,4,5)P_3_ labeling intensity, showing the effectiveness of alpelisib treatment on PI(3,4,5)P_3_ synthesis. N = 96, 128, and 109 cells for WT DMSO, WT alpelisib, KO DMSO, and KO alpelisib, respectively. K-W test: overall ∗∗∗*P* < 0.001, multiple comparisons; WT DMSO versus KO DMSO, WT DMSO versus WT alpelisib, and KO DMSO versus KO alpelisib all ∗∗∗*P* < 0.001. (**b**) Representative confocal micrographs of WT or KO HK2 cells treated with either DMSO, 10 μM-, or 50-μM alpelisib for 16 hours and then fixed with the Golgi fix and labeled using the mCh-2xFYVE PI(3)P probe (magenta) and DAPI (cyan), with quantification of the number of PI(3)P-positive puncta detected in cells treated with a range of alpelisib concentrations, as indicated, showing PI(3)P-positive punctae are reduced in a dose-responsive fashion. N = 280, 254, 210, 287, 324, 239, 340, 250, 240, and 215 cells for WT control, WT 10 μM, WT 50 μM, KO DMSO, and KO 2.5, 5, 10, 25, and 50 μM alpelisib, respectively. K-W test: overall ∗∗∗*P* < 0.001, multiple comparisons; WT DMSO versus KO DMSO, WT DMSO versus WT 10 and 50 μM alpelisib, KO DMSO versus KO 50 μM alpelisib all ∗∗∗*P* < 0.001; KO DMSO versus KO 2.5 and 5 μM alpelisib both *P* > 0.9999 (not significant [ns]); KO DMSO versus KO 10 μM alpelisib ∗∗*P* = 0.0049; KO DMSO versus KO 25 μM alpelisib ∗∗∗*P* = 0.0002. (**c**) Representative widefield micrographs of WT or KO HK2 cells treated with DMSO or 10 μM of alpelisib and then fixed with the plasma membrane fix and immunolabeled for PI(4,5)P_2_ (yellow) and DAPI (cyan), with quantification of the mean cellular PI(4,5)P_2_ labeling intensity, showing increased plasma membrane PI(4,5)P_2_ in KO cells, which is reduced by alpelisib treatment, specifically in KO cells. N = 126, 112, 85, and 116 cells for WT DMSO, WT alpelisib, KO DMSO, and KO alpelisib, respectively. K-W test: overall ∗∗∗*P* < 0.001, multiple comparisons; WT DMSO versus KO DMSO and KO DMSO versus KO alpelisib both ∗∗∗*P* < 0.001; WT DMSO versus WT alpelisib *P* = 0.4752 (ns). (**d**) Representative widefield micrographs of WT or KO HK2 cells treated with DMSO or 10 μM of alpelisib and then fixed with the 4% formaldehyde fix and immunolabeled for PI(4,5)P_2_ (yellow) and DAPI (cyan), with quantification of the number of PI(4,5)P_2_-positive puncta, showing increased PI(4,5)P_2_ puncta in KO cells, which is reduced by alpelisib treatment, specifically in KO cells. N = 75, 65, 52, and 69 cells for WT DMSO, WT alpelisib, KO DMSO, and KO alpelisib, respectively. K-W test: overall ∗∗∗*P* < 0.001, multiple comparisons; WT DMSO versus KO DMSO and KO DMSO versus KO alpelisib both ∗∗∗*P* < 0.001; WT DMSO versus WT alpelisib *P* > 0.99 (ns). a.u., arbitrary units. To optimize viewing of this image, please see the online version of this article at www.kidney-international.org.

To examine how phosphoinositide lipids are affected by *OCRL* KO and alpelisib treatment, we stained HK2 cells with antibodies or protein domains against PI(3,4,5)P_3_, PI(3)P, and PI(4,5)P_2_, using optimized fixation conditions for the plasma membrane or intracellular staining.^24^^,^^25^ PI(3,4,5)P_3_ has a diffuse localization to the plasma membrane, and alpelisib treatment (10 μM) induced a robust decrease in staining for PI(3,4,5)P_3_ in both WT and *OCRL* KO cells, as expected from its mechanism of action and specificity for class IA PI3K p110α (Figure 3a). PI(3,4,5)P_3_ is expected to be dephosphorylated progressively as membranes are endocytosed and trafficked into endosomes.^14^^,^^26^^,^^27^ By fixing cells such that intracellular PI(3)P is preserved,^25^ we found that PI(3)P punctae were also reduced by alpelisib treatment (in a dose-responsive fashion apparent from concentrations as low as 10 μM for the 16-hour treatment) in both WT and *OCRL* KO cells (Figure 3b and Supplementary Figure S2C). As expected, the KO of *OCRL* in HK2 cells induced a marked increase in the levels of PI(4,5)P_2_ both at the plasma membrane (Figure 3c) and intracellularly (Figure 3d), compared with WT cells.^10^^,^^28^ Treatment with alpelisib markedly reduced the elevation of PI(4,5)P_2_ generated by *OCRL* KO in both compartments, whereas it had no effect in WT cells (Figure 3c and d). Alpelisib is reported to inhibit PI 4-kinase β with a 50% inhibitory concentration of 0.5 μM,^19^ which is a possible source of the reduction in PI(4,5)P_2_. Collectively, our data suggested that alpelisib inhibits actin assembly on *OCRL* KO endosomes via a bispecific effect on the PI(4,5)P_2_ and PI(3)P levels.

### Alpelisib reduces intracellular phosphoinositides and alleviates cytoskeletal defects in *Ocrl*^*Y/−*^ mPTCs

**Figure 4:**
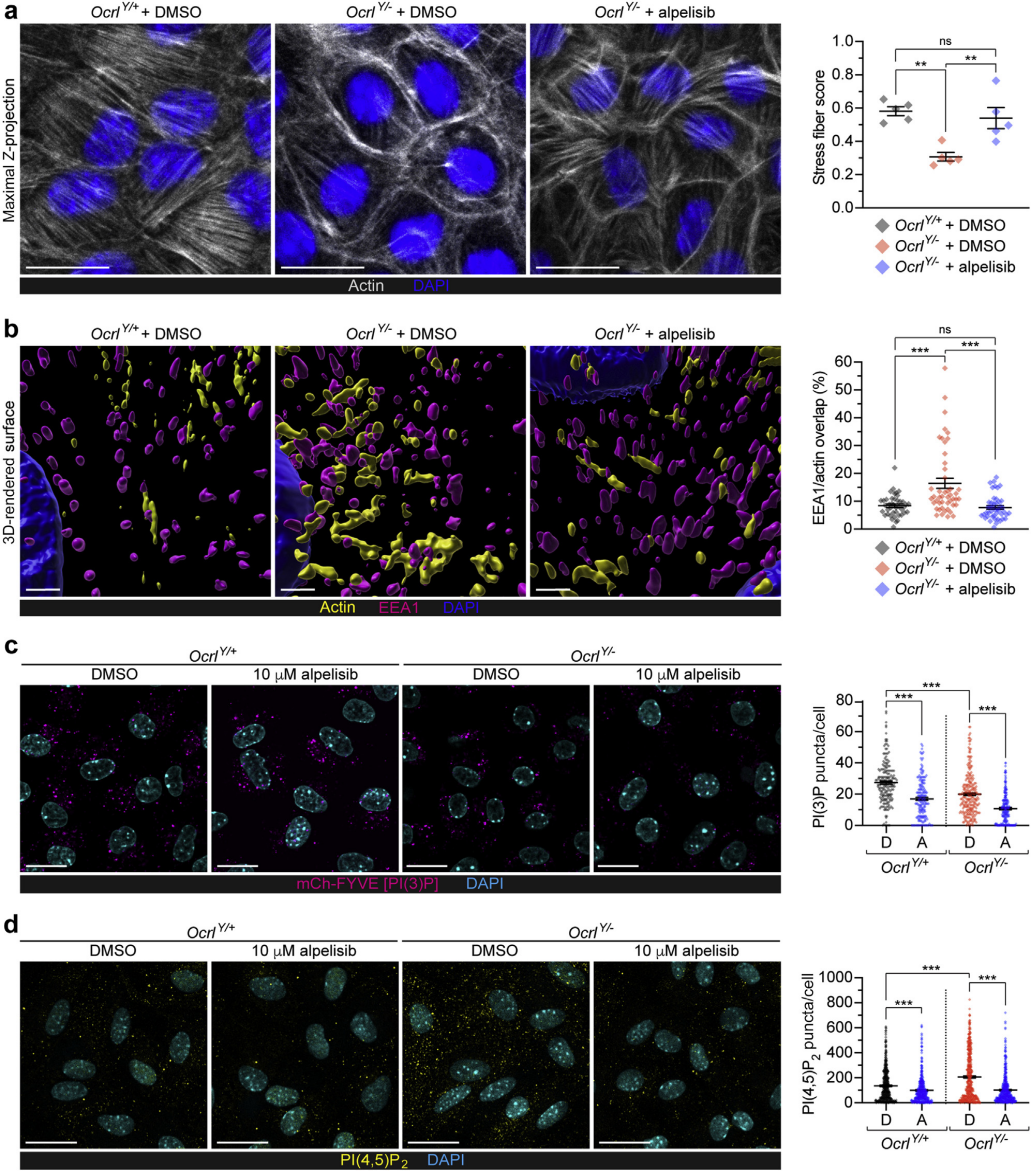
**Alpelisib alleviates actin defects of *Ocrl* in cultured humanized *Ocrl***^***Y/−***^**mouse PTCs (mPTCs).** (**a**) Representative maximum intensity Z-projection confocal micrographs of *Ocrl*^*Y/+*^ or *Ocrl*^*Y/−*^ mPTCs treated with dimethylsulfoxide (DMSO) or 10 μM of alpelisib for 16 hours and then fixed with the 4% formaldehyde fix and immunolabeled for actin (phalloidin) (white) and 4′,6-diamidino-2-phenylindole (DAPI) (blue), with quantification of the degree to which stress fibers are present in each condition, showing that stress fibers lost in *Ocrl*^*Y/−*^ mPTCs are rescued by alpelisib treatment. The lines indicate means ± SEM, and the data points indicate each imaging region: N = 5 imaging regions per condition (each containing approximately 15–20 cells). Significance was assessed by ordinary 1-way analysis of variance (ANOVA) with Holm-Sidak’s multiple comparison test, ∗∗*P* = 0.001, multiple comparisons; *Ocrl*^*Y/+*^ DMSO versus *Ocrl*^*Y/−*^ DMSO ∗∗*P* = 0.002, *Ocrl*^*Y/−*^ DMSO versus *Ocrl*^*Y/−*^ alpelisib ∗∗*P* = 0.004, *Ocrl*^*Y/+*^ DMSO versus *Ocrl*^*Y/−*^ alpelisib *P* = 0.50 (not significant [ns]). Bars = 20 μm. (**b**) High-magnification representative 3D surface renderings of *Ocrl* mPTCs treated with DMSO or 10 μM of alpelisib for 16 hours and then fixed with the 4% formaldehyde fix and immunolabeled for early endosome antigen 1 (EEA1) (purple), actin (phalloidin, yellow), and DAPI (blue), and quantification illustrating rescue of the actin-endosomal overlap by alpelisib. Lower-magnification views indicating overviews of these regions are shown in Supplementary Figure S4B and Supplementary Movies S1–S3. Lines indicate mean ± SEM. N = 42, 47, and 45 randomly selected fields for *Ocrl*^*Y/+*^ + DMSO, *Ocrl*^*Y/−*^ + DMSO, and *Ocrl*^*Y/−*^ + alpelisib conditions, respectively, in each case pooled from 4 mouse kidneys per condition. Significance was tested by Kruskal-Wallis (K-W) ANOVA with Dunn’s multiple comparisons test: overall ∗∗∗*P* < 0.001, multiple comparisons; *Ocrl*^*Y/+*^ DMSO versus *Ocrl*^*Y/−*^ DMSO and *Ocrl*^*Y/−*^ DMSO versus *Ocrl*^*Y/−*^ alpelisib ∗∗∗*P* < 0.001; *Ocrl*^*Y/+*^ DMSO versus *Ocrl*^*Y/−*^ alpelisib *P* > 0.99 (ns). Bars = 1 μm. (**c**) Representative confocal micrographs of *Ocrl*^*Y/+*^ or *Ocrl*^*Y/−*^ mPTCs treated with DMSO or 10-μM alpelisib and then fixed with the Golgi fix and labeled using the mCh-2xFYVE PI(3)P probe (magenta) and DAPI (cyan), with quantification of the number of PI(3)P-positive puncta detected in cells. N = 188, 177, 246, and 206 cells from *Ocrl*^*Y/+*^ + DMSO, *Ocrl*^*Y/+*^ + alpelisib, *Ocrl*^*Y/−*^ + DMSO, and *Ocrl*^*Y/−*^ + alpelisib, respectively; cells were pooled from 3 *Ocrl* kidneys per group. K-W test: overall ∗∗∗*P* < 0.001, multiple comparisons; *Ocrl*^*Y/+*^ DMSO versus *Ocrl*^*Y/−*^ DMSO, *Ocrl*^*Y/+*^ DMSO versus *Ocrl*^*Y/+*^ alpelisib, and *Ocrl*^*Y/−*^ DMSO versus *Ocrl*^*Y/−*^ alpelisib, all ∗∗∗*P* < 0.001. Bars = 20 μm. (**d**) Representative confocal micrographs of *Ocrl*^*Y/+*^ or *Ocrl*^*Y/−*^ mPTCs treated with DMSO or 10 μM of alpelisib for 16 hours then fixed with the 4% formaldehyde fix and immunolabeled for phosphatidylinositol (PI) 4,5-bisphosphate [PI(4,5)P_2_] (yellow) and DAPI (cyan), with quantification of the number of PI(4,5)P_2_-positive puncta, showing increased PI(4,5)P_2_ puncta in *Ocrl*^*Y/−*^ cells, which is reduced by alpelisib treatment. N = 477, 444, 423, and 494 from *Ocrl*^*Y/+*^ + DMSO, *Ocrl*^*Y/+*^ + alpelisib, *Ocrl*^*Y/−*^ + DMSO, and *Ocrl*^*Y/−*^ + alpelisib, respectively; cells were pooled from 3 *Ocrl* kidneys per group. K-W test: overall ∗∗∗*P* < 0.001, multiple comparisons; *Ocrl*^*Y/+*^ DMSO versus *Ocrl*^*Y/−*^ DMSO, *Ocrl*^*Y/+*^ DMSO versus *Ocrl*^*Y/+*^ alpelisib, and *Ocrl*^*Y/−*^ DMSO versus *Ocrl*^*Y/−*^ alpelisib, all ∗∗∗*P* < 0.001. Bars = 20 μm. To optimize viewing of this image, please see the online version of this article at www.kidney-international.org.

We next tested whether alpelisib had the same effects in primary cells of PT cells derived from the humanized *Ocrl*-deficient mouse model (*Ocrl*^*Y/−*^), which recapitulates the abnormal actin polymerization and endocytic defect observed in patient-derived cells.^10^^,^^11^ The mouse PTCs (mPTCs) expressed similar class I PI3K regulatory subunits to HK2 cells in both WT and *Ocrl*^*Y/−*^ mice, and displayed a similar toxicity profile toward alpelisib when assessed by MTT assays (Supplementary Figure S3). Imaging by confocal microscopy revealed a striking disruption of the normal F-actin stress fiber architecture in mPTCs from *Ocrl*^*Y/−*^ compared with *Ocrl*^*Y/+*^ mice, likely due to the relocation of actin regulatory proteins from their normal cellular locations to endosomes, as large assemblies of actin surround these organelles and are thought to be the origin of the trafficking defect.^10^^,^^29^ Treatment of *Ocrl*^*Y/−*^ mPTCs with alpelisib (10 μM) restored the stress fiber architecture (Figure 4a).

As the main problem in Lowe syndrome and Dent disease 2 is reduced endosomal trafficking and consequent megalin degradation, we sought to distinguish endosomal actin from the stress fibers. We collected laser-scanning confocal z-stacks throughout the cells, reconstructed the staining pattern in 3D surface renderings, and used size shape/parameters to identify punctate actin structures and the degree to which they overlapped with endosomal structures (Supplementary Figure S4 and Movies S1–S3). This analysis revealed that treatment with alpelisib decreased aberrant polymerization of F-actin at endosomes stained with EEA1, distinct from the stress fiber architecture (Figure 4b). The mPTCs derived from *Ocrl*^*Y/−*^ mice showed significant changes in intracellular staining for PI(3)P (decreased) and PI(4,5)P_2_ (increased), compared with mPTCs from WT *Ocrl*^*Y/+*^ mice (Figure 4c and d). Treatment of mPTCs from *Ocrl*^*Y/−*^ mice with alpelisib resulted in a significant reduction in intracellular PI(3)P and PI(4,5)P_2_ staining (Figure 4c and d).

### Alpelisib improves endocytic uptake in *Ocrl*^*Y/−*^ mPTCs

**Figure 5:**
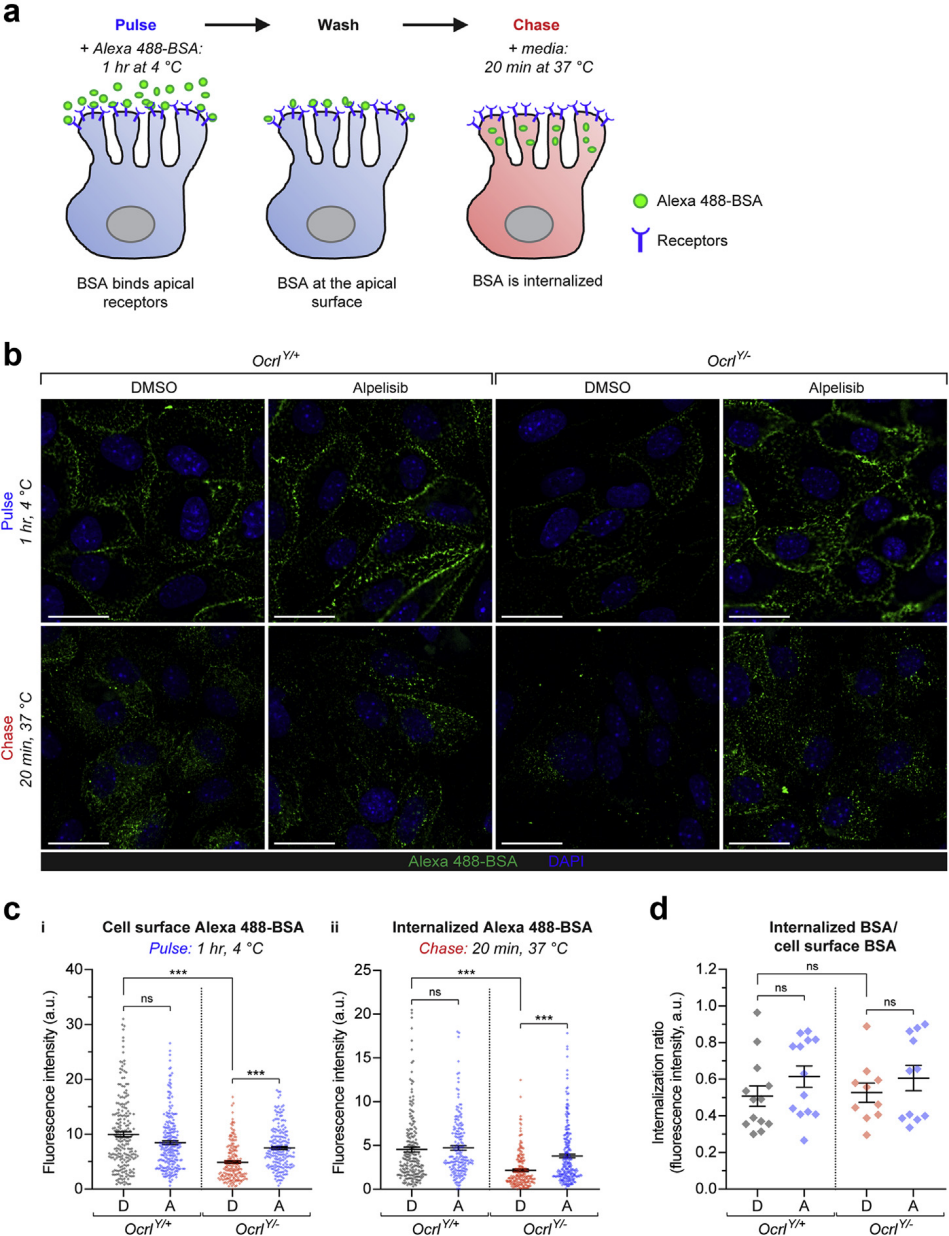
**Alpelisib improves endocytic uptake of humanized *Ocrl***^***Y/−***^**mouse PTCs (mPTCs).** (**a**) Schematic illustrating the pulse-chase experiment used to examine the binding and internalization of Alexa 488-bovine serum albumin (BSA) into mPTCs. Cells were exposed to Alexa 488-BSA (0.2 mg/ml) for 1 hour at 4 °C to allow BSA to bind to cell surface receptors (pulse) and then warmed to 37 °C in cell medium for 20 minutes before fixation to allow ligand internalization (chase). (**b**) Representative confocal micrographs of *Ocrl*^*Y/+*^ or *Ocrl*^*Y/−*^ mPTCs treated with dimethylsulfoxide (DMSO) or 10 μM of alpelisib for 16 hours and subjected to the Alexa 488-BSA (green) pulse-chase experiment, before being fixed and labeled for 4′,6-diamidino-2-phenylindole (DAPI) (blue). The pulse phase of the experiment is shown in the top panel for each condition, with the chase below. Bars = 20 μm. (**c**) Quantification of cell surface Alexa 488-BSA (*i*) and internalized Alexa 488-BSA (*ii*), evaluated as mean fluorescence intensities per cell. Alpelisib rescues the rescued BSA binding and internalization observed in *Ocrl*^*Y/−*^*cells.* N = 209, 228, 186, and 196 cells for *Ocrl*^*Y/+*^ DMSO, *Ocrl*^*Y/+*^ alpelisib, *Ocrl*^*Y/−*^ DMSO, and *Ocrl*^*Y/−*^ alpelisib conditions, respectively in (*i*) and N = 203, 183, 199, and 233 cells for *Ocrl*^*Y/+*^ DMSO, *Ocrl*^*Y/+*^ alpelisib, *Ocrl*^*Y/−*^ DMSO, and *Ocrl*^*Y/−*^ alpelisib conditions, respectively in (*ii*), in each case pooled from 2 mouse kidneys per condition; each data point represents the mean fluorescence intensity in an individual cell. Significance was tested by Kruskal-Wallis (K-W) analysis of variance (ANOVA) with Dunn’s multiple comparisons test: for (*i*) cell surface BSA, overall ∗∗∗*P* < 0.001, multiple comparisons: *Ocrl*^*Y/+*^ DMSO versus *Ocrl*^*Y/−*^ DMSO and *Ocrl*^*Y/−*^ DMSO versus *Ocrl*^*Y/−*^ alpelisib ∗∗∗*P* < 0.001; *Ocrl*^*Y/+*^ DMSO versus *Ocrl*^*Y/+*^ alpelisib *P* = 0.60 (not significant [ns]); for (*ii*) internalized BSA, overall ∗∗∗*P* < 0.001, multiple comparisons: *Ocrl*^*Y/+*^ DMSO versus *Ocrl*^*Y/−*^ DMSO and *Ocrl*^*Y/−*^ DMSO versus *Ocrl*^*Y/−*^ alpelisib ∗∗∗*P* < 0.001; *Ocrl*^*Y/+*^ DMSO versus *Ocrl*^*Y/+*^ alpelisib *P* = 0.66 (ns). (**d**) Quantification of the ratio between the cell surface Alexa 488-BSA and the internalized Alexa 488–BSA fluorescence intensities, showing no significant differences in ratios between each condition. Each point represents the average of the ratio in a field containing approximately 15–20 cells (N = 13, 13, 10, and 11 randomly selected fields for *Ocrl*^*Y/+*^ DMSO, *Ocrl*^*Y/+*^ alpelisib, *Ocrl*^*Y/−*^ DMSO, and *Ocrl*^*Y/−*^ alpelisib conditions, respectively, in each case pooled from 2 mouse kidneys per condition). Significance was tested by K-W ANOVA with Dunn’s multiple comparisons test: overall *P* = 0.46 (ns), multiple comparisons: *Ocrl*^*Y/+*^ DMSO versus *Ocrl*^*Y/−*^ DMSO and *Ocrl*^*Y/−*^ DMSO versus *Ocrl*^*Y/−*^ alpelisib *P* > 0.99 (ns); *Ocrl*^*Y/+*^ DMSO versus *Ocrl*^*Y/+*^ alpelisib *P* = 0.84 (ns). To optimize viewing of this image, please see the online version of this article at www.kidney-international.org.

We next assessed whether the effect of alpelisib on the cytoskeletal and vesicular defects observed in mPTCs resulted in changes in the endocytic uptake capacity. To differentiate the effect of alpelisib on binding and/or internalization of the ligand, mPTCs were first incubated with labeled bovine serum albumin (BSA), to induce binding of the probe with the endocytic receptors, and then incubated with growth medium, to follow the internalization of albumin (Figure 5a). The *Ocrl*^*Y/−*^ cells showed a lower plasma membrane binding of Alexa 488-bovine serum albumin along with impaired internalization of albumin compared with *Ocrl*^*Y/+*^ cells. Treatment with alpelisib rescued both the binding and the uptake of albumin in the *Ocrl*^*Y/−*^ cells, with a 50% overall rescue of endocytic uptake (Figure 5b and c). These data were supported by the measurement of total albumin uptake (Supplementary Figure S5a and b). We noted that the internalized/bound Alexa 488-bovine serum albumin ratio remained similar between the different conditions (Figure 5d), implying that the reduced uptake of albumin is mainly due to impaired binding rather than a defective internalization process *per se.*

### Alpelisib improves PT dysfunction and receptor-mediated endocytosis in *Ocrl*^*Y/−*^ mice

**Figure 6:**
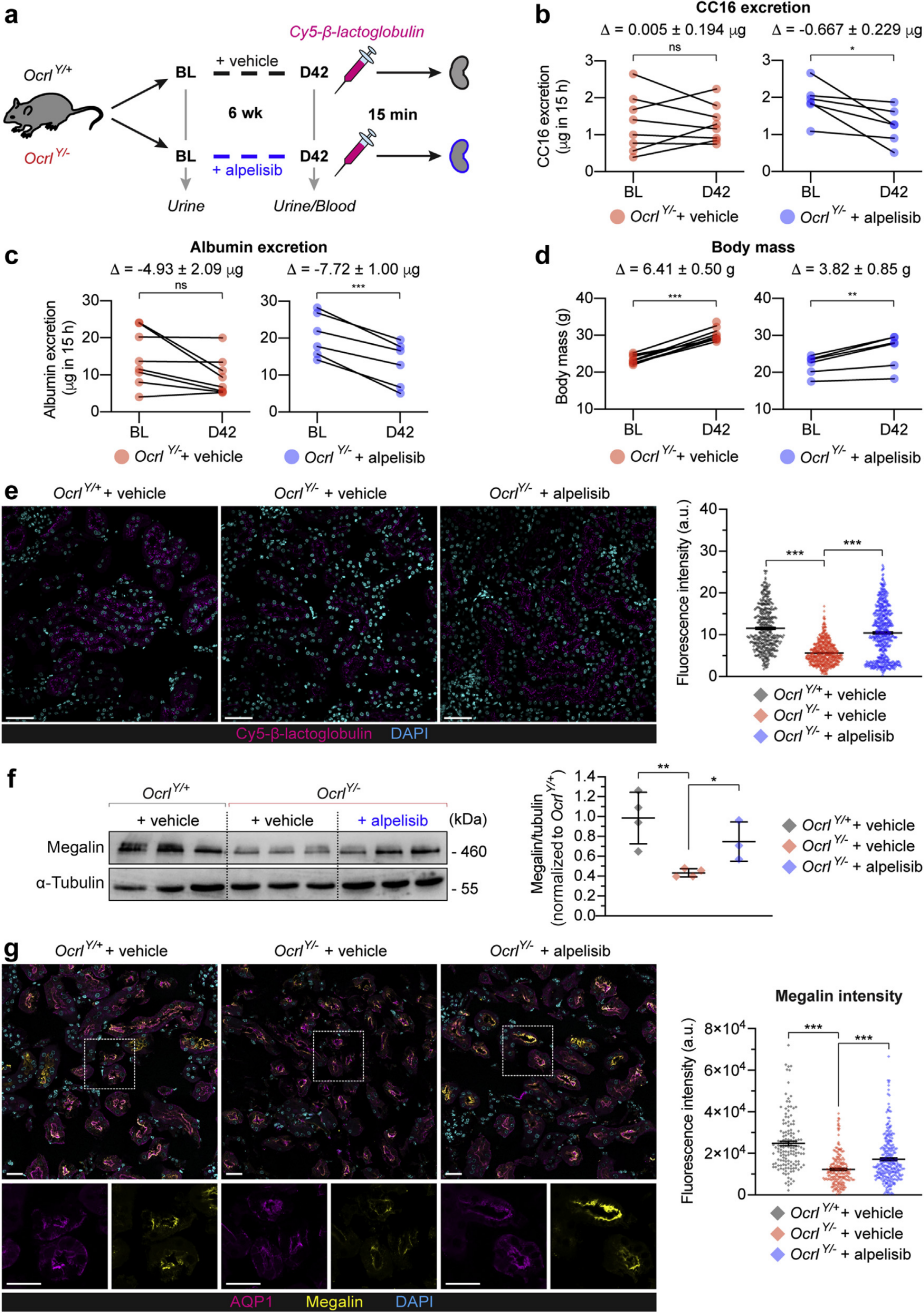
**Alpelisib improves the proximal tubule (PT) function of *Ocrl***^***Y/*−**^**mice, restoring ligand uptake and megalin expression.** (**a**) Experimental setup. *Ocrl* mice were treated for 6 weeks with a daily oral dose of either carboxymethylcellulose 1% (vehicle) or alpelisib (50 mg/kg body weight). On the last day of the treatment, mice were injected with Cy5-labeled β-lactoglobulin (N = 6 *Ocrl*^*Y/−*^, or 8 *Ocrl*^*Y/−*^ mice per group). (**b–d**) The Δ values shown indicate the mean change from BL to D42 for the condition ± SEM. (**b**) Clara cell protein 16 (CC16), (**c**) albumin urinary output (both within 15 hours), and (**d**) body mass was measured in *Ocrl*^*Y/−*^ mice treated with either vehicle or alpelisib at the indicated time point. Each dot represents 1 mouse. Significance was assessed by 2-tailed paired Student’s *t* test; in (**b**) CC16 output change relative to baseline; + vehicle, *P* = 0.9802 (not significant [ns]), + alpelisib, ∗*P* = 0.0334; in (**c**) albumin output change relative to baseline; + vehicle, *P* = 0.0502 (ns), + alpelisib, ∗∗∗*P* = 0.0006; in (**d**) body mass change relative to baseline; + vehicle, ∗∗∗*P* < 0.0001, + alpelisib, ∗∗*P* = 0.0083. (**e**) Representative confocal micrographs showing Cy5-labeled β-lactoglobulin (magenta) 15 minutes after tail vein injection and labeled for 4′,6-diamidino-2-phenylindole (DAPI) (cyan), plus quantification of the corresponding fluorescent signals from *Ocrl* mouse kidneys (N = 412, 500, and 588 tubules, respectively, for *Ocrl*^*Y/+*^+ vehicle, *Ocrl*^*Y/−*^ + vehicle, and *Ocrl*^*Y/−*^ + alpelisib) for 3 mice per treatment group. β-Lactoglobulin uptake is rescued by alpelisib treatment. Bars = 20 μm. In the quantifications, each dot represents fluorescence intensity normalized by tubule area; plotted data indicate the mean ± SEM. Significance was assessed by the Kruskal-Wallis (K-W) test followed by Dunn’s multiple comparison test; ∗∗∗*P* < 0.001, multiple comparisons; *Ocrl*^*Y/+*^+ vehicle versus *Ocrl*^*Y/−*^ + vehicle and *Ocrl*^*Y/−*^ + vehicle versus *Ocrl*^*Y/−*^ + alpelisib, both ∗∗∗*P* < 0.001. (**f**) Western blotting and densitometry analysis of megalin levels in whole kidney lysates from *Ocrl* mice. α-Tubulin was used as a loading control. The reduced megalin expression in *Ocrl*^*Y/−*^ is rescued by alpelisib. In the quantification of the densitometry analysis, each dot represents 1 mouse (N = 4 *Ocrl*^*Y/+*^+ vehicle and *Ocrl*^*Y/−*^ + vehicle and N = 3 *Ocrl*^*Y/−*^ + alpelisib mice); lines indicate mean ± SEM. Significance was assessed by 2-tailed unpaired Student’s *t* tests: *Ocrl*^*Y/+*^+ vehicle versus *Ocrl*^*Y/−*^ + vehicle; ∗∗*P* = 0.0057, *Ocrl*^*Y/−*^ + vehicle versus *Ocrl*^*Y/−*^ + alpelisib, ∗*P* = 0.0246. (**g**) Representative confocal micrographs with high-magnification insets and quantification of megalin (yellow) intensity in AQP1^+^ PTs (magenta) from *Ocrl* kidneys also labeled for DAPI (cyan) illustrating rescue of megalin levels after alpelisib treatment. Bars = 20 μm. In the quantifications, each dot represents fluorescence intensity normalized by tubule area; plotted data indicate the mean ± SEM; N = 142, 191, and 266 tubules, respectively, for *Ocrl*^*Y/+*^+ vehicle, *Ocrl*^*Y/−*^ + vehicle, and *Ocrl*^*Y/−*^ + alpelisib for 3 mice per treatment group. Significance was assessed by K-W analysis of variance followed by Dunn’s multiple comparison test; overall ∗∗∗*P* < 0.001, multiple comparisons; *Ocrl*^*Y/+*^+ vehicle versus *Ocrl*^*Y/−*^ + vehicle and *Ocrl*^*Y/−*^ + vehicle versus *Ocrl*^*Y/−*^ + alpelisib, both ∗∗∗*P* < 0.001. To optimize viewing of this image, please see the online version of this article at www.kidney-international.org.

We tested the potential therapeutic effect of alpelisib on PT dysfunction *in vivo*. *Ocrl* mice were administered with either vehicle or alpelisib (50 mg/kg body weight per day) by oral gavage for 6 weeks (Figure 6a). This regimen was chosen according to previous *in vivo* studies demonstrating efficacy and safety.^22^^,^^30^ In line with alpelisib administration being known to cause insulin resistance and hyperglycemia,^22^ alpelisib treatment led to a significant increase in glycosuria in both *Ocrl* ^*Y/+*^ and *Ocrl* ^*Y/−*^ mice, which could be considered as a biomarker for drug dosing (Supplementary Table S1). After 6 weeks of alpelisib treatment, the *Ocrl*^*Y/−*^ mice displayed a significant reduction in the urinary excretion of the LMW proteins CC16 (−34%) and albumin (−38%), compared with vehicle-treated controls (Figure 6b and c), whereas volume of urine and other parameters were unaffected (Supplementary Table S1). Alpelisib-treated mice of both genotypes showed a similar reduction in growth rate relative to control mice, as previously described.^22^ Nevertheless, both vehicle and treated mice gained body weight, indicating that the drug does not severely affect postnatal development (Figure 6d and Supplementary Table S1).

To test whether the effect of alpelisib on LMW proteinuria reflected recovery of receptor-mediated endocytosis in PT cells, we followed the *in vivo* uptake of Cy5-labeled β-lactoglobulin in a mouse kidney. Alpelisib-treated *Ocrl*^*Y/−*^ mice displayed a significant rescue of Cy5-labeled β-lactoglobulin uptake, close to the extent of internalization observed in vehicle-treated *Ocrl*^*Y/+*^ mice (Figure 6e). This was mirrored by a significant rescue in expression of the endocytic receptor megalin in PT cells, as observed by immunolabeling and western blotting of the kidney lysates of alpelisib-treated *Ocrl*^*Y/−*^ mice; note that there was no change in expression of the PT marker AQP1 (Figure 6f and g and Supplementary Figure S5C and D). These data show that the PI3K inhibitor alpelisib induces a substantial improvement of the PT endocytic machinery and reduces LMW proteinuria in a humanized mouse model for Lowe syndrome.

## Discussion

**Figure 7:**
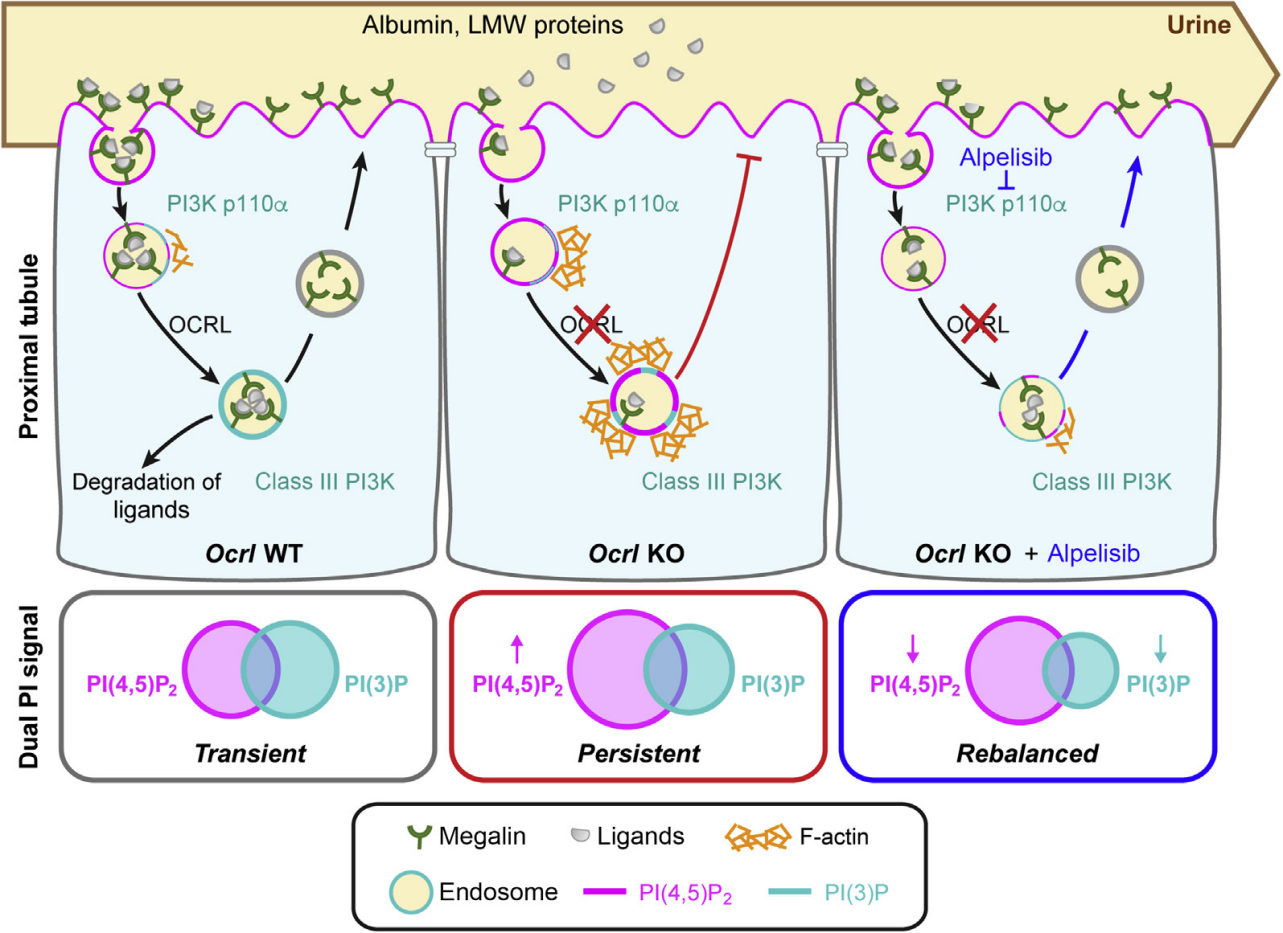
**Proposed model depicting the therapeutic effect of alpelisib on proximal tubule cells in Lowe syndrome.** Proximal tubule cells reabsorb urinary ligands (e.g., albumin and low-molecular-weight proteins) through megalin-mediated endocytosis. The 5-phosphatase activity of OCRL regulates the transition from high phosphatidylinositol (PI) 4,5-bisphosphate [PI(4,5)P_2_] at the plasma membrane to low levels at the early endosome with a transient coincidence of PI(4,5)P_2_ and PI(3)P in the vesicles. Once in the early endocytic compartment, the ligands dissociate from the receptors and are delivered to the lysosome for degradation, whereas the receptors recycle back to the plasma membrane for a new cycle of cargo binding. The loss of OCRL leads to an ectopic accumulation of PI(4,5)P_2_ at the endosomal compartment, which results in a persistent coincidence with PI(3)P. We suggest that this event is responsible for aberrant F-actin polymerization blocking endocytic recycling and preventing ligand reabsorption (middle panel). Alpelisib rebalances the levels of  PI(4,5)P_2_ and PI(3)P, which results in decreased actin polymerization and improvement of the endocytic machinery and absorptive capacity of proximal tubule cells (right panel).

There is currently no treatment to alleviate the defective endocytosis causing PT dysfunction in Lowe syndrome and Dent disease 2. Our findings reveal that alpelisib alleviates the aberrant actin phenotype by reducing levels of PI(4,5)P_2_ and PI(3)P, causing a substantial improvement of the endocytic machinery and absorptive capacity in cellular systems and a humanized mouse model for Lowe syndrome/Dent disease 2. These results support the link between phosphoinositide lipids, actin polymerization, and endocytic trafficking, with immediate relevance for highly active epithelial cells involved in crucial homeostatic processes (Figure 7). Given the lack of effective therapies and the apparent safety of this class of PI3K inhibitors, alpelisib is a promising candidate for drug repurposing in Lowe syndrome and Dent disease.

LMW proteinuria is the most consistent feature encountered in patients with Lowe syndrome and Dent disease 2 due to inactivating mutations in *OCRL*.^4^ These LMW proteins can readily be detected and quantified, offering a faithful biomarker of defective receptor-mediated endocytosis in PT cells.^1^ LMW proteinuria is particularly relevant, as it is detected more consistently and often earlier than other solutes (e.g., glucose, phosphate, amino acids) being part of the classical “renal Fanconi syndrome”—at least in congenital disorders of the endolysosomal pathway.^1^ Here, we show that alpelisib rescues the apical endocytic uptake capacity of PT cells *in vitro* and *in vivo*, due to restored levels of megalin receptor at the plasma membrane. This effect is reflected by significant reductions in the urinary loss of LMW proteins (CC16 and albumin) in *Ocrl*^*Y/−*^ mice treated with alpelisib for 6 weeks. These effects of alpelisib have been observed in mPTCs, which keep their apical differentiation and are particularly well suited to investigate receptor-mediated endocytosis in physiology and disease.^9^ In particular, we obtained mPTCs from a humanized mouse model expressing human INPP5B in *Ocrl*^*Y/−*^;*Inpp5b*^*−/−*^ background, which allows us to investigate the specific consequences of the loss of OCRL activity.^11^^,^^23^

The rescue of endocytosis by alpelisib is explained by its effect on actin machinery, clearly evidenced in *OCRL* KO HK2 cells and mPTCs. Our observations thus confirm and extend previous studies showing the link between control of the phosphoinositide balance and F-actin in the early endosomal pathway in OCRL patient and KO cells.^7^^,^^10^ The functional loss of OCRL activity impairs the degradation of PI(4,5)P_2_, which, in turn, leads to a failure to uncoat clathrin-coated vesicles resulting in aberrant endosomal organelles in various cell types including the PT cells.^7^, ^8^, ^9^, ^10^ We recently showed that the excess F-actin decreases the recycling of the multiligand receptor megalin to the apical membrane of PT cells, causing defective endocytosis and LMW proteinuria.^11^

Our data indicate that the mechanism of action of alpelisib on endosomal actin arises from a bispecific effect of alpelisib inhibition: first on production of PI(3,4,5)P_3_ and its conversion to PI(3)P, and second on production of PI(4,5)P_2_. The decrease in PI(4,5)P_2_ levels directly counteracts the OCRL deficiency and is in agreement with previous work alleviating excess actin and increasing endocytic flux by reduction in the levels of phosphatidylinositol 4-phosphate 5-kinase, an enzyme that generates PI(4,5)P_2_.^10^ Alpelisib has a 0.5 μM 50% inhibitory concentration on the PI(4,5)P_2_ generating kinase PI4Kβ, which may be responsible, or it could result from more general effects on phosphoinositide balance resulting from the combination of *OCRL* KO and alpelisib treatment. We have identified that class IA PI3K inhibition by alpelisib is relevant to actin inhibition as siRNA-mediated depletion of p110α inhibits actin accumulation similarly to alpelisib treatment and copanlisib (which is not reported to inhibit PI4Ks). In RPE cells we have previously observed that siRNA of INPP4A, which converts PI(3,4)P_2_ to PI(3)P, inhibits actin assembly in *OCRL* KO cells, providing a pathway from inhibition of PI(3,4,5)P_3_ production to a decrease in endosomal PI(3)P. We showed that some PI(3)P remains, presumably the pool produced at the early endosome by class III PI3K, Vps34, because we found that endosomal trafficking is enhanced rather than suppressed by alpelisib treatment. The complex coregulation of PI metabolism and its relevance for OCRL inactivation has also been highlighted in a recent study where noncatalytic functions of phosphoinositide 3-phosphatase PTEN activates PI(4,5)P_2_ degradation via PLCXD, alleviating cellular phenotypes and absorption of ligands in a zebrafish model.^31^

The alleviation of the actin phenotype by reduced levels of PI(4,5)P_2_ and PI(3)P by alpelisib treatment agrees with the synergistic action of PI(4,5)P_2_ and PI(3)P in recruiting SNX9 to activate the actin machinery at *Ocrl* endosomes,^14^ suggesting a very effective match of alpelisib specificity to manipulate the molecular regulation of actin activation at endosomes. In turn, as previously demonstrated, reduced endosomal actin assembly in turn leads to an improvement in PT endocytosis in OCRL-deficient cells.^10^ Further characterization will be needed to better understand whether regulation of actin through phosphoinositide metabolism is the true source of the therapeutic effect that we observe with alpelisib in the *Ocrl*^*Y/−*^ mouse model. Because class I PI3K regulate pathways control cell growth, proliferation, survival, metabolism, and autophagy,^32^ we cannot exclude that the endocytic rescue is due to effects on PI(3,4,5)P_3_ directly and the combined effect on other pathways modulated by class I PI3K. More work is also needed to test whether PI3K inhibitors might also alleviate the neurologic and other clinical manifestations of patients harboring *OCRL* mutations.^4^

The recent experience of alpelisib in pediatric patients with PROS/CLOVES syndrome suggests that the drug has the potential to be well tolerated. Alpelisib is taken orally, selectively targets the α isoform of PI3K class I, and shows a minor toxicity profile compared with pan-PI3K inhibitors. As alpelisib does not completely block PI3K activity, thus maintaining functions of the signaling pathway, it may be particularly suitable for long-term use. Of interest, the hyperglycemia arising from alpelisib use may be manageable by dietary changes.^22^ Because PT dysfunction is the first manifestation of kidney disease observed in young infants with Dent disease 2 and Lowe syndrome, an early treatment of such PT dysfunction, leading to improvements in the metabolic profile and growth, might therefore slow progression to chronic kidney disease and therefore have a significant impact on lifespan and quality of life.^33^ Although the disease manifestations of *OCRL* mutation are particularly wide, recent studies based on large cohorts of genotyped patients with Lowe syndrome of Dent disease 2 did not evidence significant effects of the type of mutation or the location of the mutation on renal survival.^34^ Collectively, our data highlight the potential for repurposing alpelisib for treating PT dysfunction in Lowe syndrome/Dent disease 2, thus providing a basis for rapid and cost-effective deployment in human clinical trials.

## Methods

Full details can be found in the Supplementary Methods.

HK2 cells were treated with copanlisib, alpelisib, GSK2636771, or idelalisib at indicated concentrations for 16 hours unless otherwise stated. Proteins were extracted using standard methods from cells or kidney tissues and western blotting performed using published or commercially available antibodies. siRNA knockdown was performed using 2 transfections 72 and 24 hours before analysis. We used age- and gender-matched *Ocrl*^*Y/+*^;*Inpp5b*^*−/−*^; and *Ocrl*^*Y/−*^;*Inpp5b*^*−/−*^ mouse littermates harboring BAC-INPP5B expression. Primary cultures of mPTCs were generated from the kidneys harvested from 8 week-old *Ocrl* mice and the endocytic capacity of *Ocrl* mPTCs assessed by measuring albumin uptake. Alpelisib treatments were 10 μM for 16 hours, unless otherwise stated. Albumin uptake in mPTCs was assessed using a “pulse-chase” protocol to assess apical binding and resultant uptake. mPTCs were stained overnight with the appropriate primary antibody and for 45 minutes with suitable fluorophore-conjugated secondary antibodies and/or Alexa-488 Phalloidin. PI(3)P staining on mPTCs was performed using the FYVE domain probe. Image analysis was performed with CellProfiler, using custom pipelines to measure actin/EEA1 overlap and number of puncta, and with ImageJ to measure immunofluorescence intensity and detected edges to calculate stress fiber score. Quantitative data were expressed as means ± standard error of the mean. For the *in vivo* experiments, mice aged 6 weeks were treated with vehicle or alpelisib 50 mg/kg body weight. Urine was collected every 14 days and animals were sacrificed after 42 days of treatment, with blood and kidneys harvested. The PT endocytic capacity of *Ocrl* mice was examined by measuring β-lactoglobulin uptake.

## Disclosure

JLG has funding from AstraZeneca for a studentship in her lab. SPJ is on the advisory boards and has equity ownership of Mission Therapeutics, Carrick Therapeutics, and Adrestia Therapeutics, and is a Science Partner for Ahren Innovation Capital. All the other authors declared no competing interests.
