# Supplementary material for: The phosphoinositide 3-kinase inhibitor alpelisib restores actin organization and improves proximal tubule dysfunction in vitro and in a mouse model of Lowe syndrome and Dent disease
Source: Kidney Int. 2020 Oct;98(4):883–96. doi: 10.1016/j.kint.2020.05.040 (PMC7550850; doi:10.1016/j.kint.2020.05.040)
Supplement: Supplementary File (PDF) [file mmc1.pdf]

Supplementary Material

Phosphoinositide 3-kinase inhibitor alpelisib restores actin organization and improves proximal tubule dysfunction in Lowe syndrome and Dent disease

Marine Berquez, MSc [1,*], Jonathan R. Gadsby, PhD [2,*], Beatrice Paola Festa, PhD [1,*], Richard Butler, PhD [3], Stephen P. Jackson, PhD [2], Valeria Berno, PhD [4], Alessandro Luciani, PhD [1], Olivier Devuyst, MD, PhD [1,†], and Jennifer L. Gallop, PhD [2,†].

[1] Institute of Physiology, University of Zurich, Zurich, Switzerland.

[2] Gurdon Institute and Department of Biochemistry, Tennis Court Road, University of Cambridge, Cambridge, CB2 1QN, UK

[3] Gurdon Institute, Tennis Court Road, University of Cambridge, Cambridge, CB2 1QN, UK

[4] Experimental Imaging Center, ALEMBIC, IRCCS San Raffaele Scientific Institute, 20132, Milan, Italy

* Equal contribution; † Co-directed the study, correspondence to OD or JLG.

Supplementary Methods and References

Legends for Supplementary Videos 1-3

Supplementary Table 1

Supplementary Figure 1

Supplementary Figure 2

Supplementary Figure 3

Supplementary Figure 4

Supplementary Figure 5

Supplementary Methods

Cell culture and CRISPR KO cell line generation. Human Kidney 2 (HK2) cells (ATCC, CRL-2190) were maintained in a 37 °C, 5% CO_2 humidified incubator in DMEM/F12 media (containing GlutaMAX; Gibco, 31331) supplemented with 10% fetal bovine serum (Sigma-Aldrich) and 100 μg/ml penicillin/100 U/ml streptomycin (Gibco), and were sub-cultured twice weekly. HK2 OCRL KO cell lines were generated exactly as previously described to generate RPE-1 OCRL KO lines[1]. Briefly, the all-in-one $Cas9^{D10A}$ vector was used alongside the following single-guide RNA (sgRNA) pairs: sense sgRNA; forward (5'-3')-ACCGCCTCCTGATATCTACTGCAT, reverse (5'-3')-AAACATGCAGTAGATATCAGGAGG, antisense sgRNA; forward (5'-3')-ACCGCAAGGTTCTAACCCGCTATC, reverse (5'-3')-AAACGATAGCGGGTTAGAACCTTG, and following transfection into HK2 cells were sorted by FACS to generate monoclonal cultures, then screened by western blot to confirm *OCRL* KO.

Drug treatments in HK2 cells. Drugs were dissolved in DMSO at appropriate dilutions such that the maximum dosage added to cells was no more concentrated than a final dilution of 1:1000. Drugs were added to cells at the indicated concentrations and time points (16 hours prior to analysis, unless otherwise stated). Alpelisib and copanlisib were from Selleck chemicals (alpelisib: S2814, copanlisib: S2802). GSK2636771 and idelalisib were from Apexbio (supplied via Stratech Scientific; GSK2636771 B2186, idelalisib A3005).

siRNA treatments. Cells were grown in antibiotic free medium. siRNA was transfected into cells using Lipofectamine RNAiMAX (Thermo-Fisher) according to manufacturer's instructions. Briefly, cells were subjected to a two-shot protocol, with transfections at 72 and 24 hours prior to analysis. siRNA was added at a final concentration of 25 nM. Sequences

were from Dharmacon; p110α: ON-TARGETplus Human PIK3CA (J-003018-15), scramble: siGENOME Non-Targeting siRNA #2 (D-001210-02).

Mouse models. Experiments were conducted on age- and gender-matched *Ocrl*$^{Y/+}$;*Inpp5b*$^{-/-}$; and *Ocrl*$^{Y/-}$;*Inpp5b*$^{-/-}$ mouse littermates harbouring BAC-INPP5B expression (129S/SvEv * 129S6/SvEvTac * FVB/N* C57BL/6 background)[2]. Mice aged 6 weeks were treated with vehicle (1% carboxymethylcellulose, n=8 mice per genotype) or with alpelisib (MedChem Express; 50 mg/kg body weight in 1% carboxymethylcellulose, n=8 mice per genotype) daily by oral gavage and sacrificed after 42 days of treatment. Urine samples were collected every 14 days. Blood and kidneys were harvested at the time of sacrifice. During the treatment, two *Ocrl*$^{Y/-}$ mice treated with alpelisib were injured by the oral gavage, euthanized and excluded from the analysis. All of the experiments were performed in accordance with the ethical guidelines at University of Zurich and the legislation of animal care and experimentation of Canton Zurich, Switzerland (ZH039/19).

Renal function parameters and glycaemia. Urine and blood parameters were measured as described[2, 3]. The urinary excretion values were obtained from overnight (15 hours) urine collection. Plasma glucose was analysed using GLU-TEST STRIPS (Nova Biomedical, 42214) according to manufacturer's instructions.

Primary cultures of mouse proximal tubule cells. The kidneys were harvested from *Ocrl* mice (8 weeks) and used to generate primary cultures of mPTCs as previously described[3]. Where indicated, the cells were treated with alpelisib (10 µM for 16 h, unless otherwise stated). The cells were processed and analysed as described below.

Cell viability assay. The viability of HK2 cells and mPTCs after drug treatments were assessed via MTT assay (ab211091, Abcam) in accordance with the manufacturer's

instructions. Briefly, the cells were washed three times with PBS and then incubated with 0.5 mg/ml of MTT diluted in medium. After 4 hours of incubation at 37 °C and until appearance of intracellular purple formazan crystals, the remaining crystals were dissolved with dimethyl sulfoxide (276855, Sigma-Aldrich) or SDS, and the absorbance measured at 570 nm.

Immunofluorescence.

HK2 cells were grown on glass coverslips. The kidneys and mPTCs were harvested and processed as previously described[2, 3]. Samples were fixed using one of the following fixation methods (typically the 4% formaldehyde fix, unless otherwise stated). Washes were performed between each step in all of the protocols.

1. <u>4% formaldehyde fix</u>

Samples were fixed in 4% formaldehyde and permeabilised in permeabilization/blocking buffer for 20 minutes (0.1% saponin, 50 mM NH_4Cl, 10% goat serum (HK2 cells) or 0.5% BSA (mouse samples) in PBS). Samples were stained for either 1 hour (HK2 cells) or overnight (kidneys and mPTCs) with primary antibodies, and for 45 minutes with secondary antibodies, then with DAPI for 5 minutes prior to mounting.

2. <u>Plasma membrane fix</u>

This was specifically used to preserve and examine plasma membrane labelling of $PI(4,5)P_2$ and $PI(3,4,5)P_3$ in HK2 cells, based on methods of Hammond et al[4]. Samples were fixed in 4% formaldehyde + 0.2% glutaldehyde for 15 minutes. After fixation, samples were transferred to 4 °C (and were kept at this temperature until the post fixation step). They were blocked and permeabilized using a buffer containing 10% goat serum and 0.5% saponin in buffer A (150 mM NaCl, 20 mM Na-HEPES, pH 7.4, 2 mM EDTA) for 45 minutes. Primary antibodies were made up in antibody incubation solution (1% goat serum and 0.1% saponin in buffer A), and incubated with the samples for 1 hour. Secondary antibodies were incubated with the sample for 45 minutes, followed by DAPI for 5 minutes prior to mounting.

Cells were then subjected to a post-fixation step in 2% formaldehyde, which was added at 4 ^{o}C for 5 minutes, followed by 10 minutes warming to room temperature before mounting.

3. Golgi fix

This was specifically used to preserve PI(3)P staining on endosomes, and was modified from previously established protocols[1, 5] . Solutions were made up in buffer A. Briefly, cells were fixed in 2% formaldehyde for 15 mins, after which they were permeabilized using 20 μM digitonin. Cells were blocked in 10% goat serum containing 1 μg/ml purified mCh-2xFYVE, then immunolabelled using an anti-RFP primary antibody and DAPI. Cells were post-fixed in 2% formaldehyde for five minutes prior to mounting.

The following antibodies and other fluorescent reagents were used; mCherry-2xFYVE[1], mouse anti-PI(4,5)P_2 (abcam ab11039; used in HK2 cells), mouse anti-PI(4,5)P_2 (Echelon Z-A045; used in mPTCs), mouse anti-PI(3,4,5)P_3 (Echelon, Z-A345), mouse anti-EEA1 (BD Bioscience, 610456), sheep anti-megalin (gift from P. Verroust and R. Kozyraki, INSERM, Paris, France), rabbit anti-AQP1 (ab2219, Millipore), rabbit anti-RFP (Rockland, 600-401-379), Alexa-Fluor conjugated secondary antibodies (Goat anti rabbit AF 568, A11011, Goat anti mouse 647 A21235, Thermo-Fisher), Alexa-488 Phalloidin (A12379, Thermo-Fisher), and DAPI (62247, Thermo-Fisher). Samples were mounted with either hydromount (National Diagnostics, in HK2 cells) or Prolong Gold Anti-fade reagent (mouse samples).

Microscopy.

HK2 cell EEA1/actin colocalization images were acquired on a Zeiss LSM 880 equipped with Airyscan and Plan-Apochromat 40x NA 1.3 or Plan-Apochromat 63x NA 1.4 objectives using the Airyscan Fast imaging mode. Images were processed using Airyscan processing, strength 6. HK2 cell confocal images were taken on an inverted microscope (Ti-E; Nikon) equipped with a 250-μm piezo-driven Z stage/controller (NanoScanZ) as an 11 x 0.4 μm Z-stack using a spinning disk unit (X-light Nipkow; Crest) and LED illumination (Lumencor Spectra X) through a Plan Apo 60x 1.4 NA oil objective (Nikon). Images were captured on

an EMCCD camera (Evolve Delta; Photometrics) in 16-bit depth using Metamorph (software version 7.8.2.0). HK2 cell widefield images were taken on an inverted microscope (Ti-E; Nikon) through a Plan Apo 60x 1.4 NA oil objective (Nikon). Images were captured on an EMCCD camera (iXon Ultra 897; Andor) in 16-bit depth using Nikon NIS-Elements AR (software version 4.50). All mPTCs and mouse tissue images were obtained using either a Leica SP5 confocal laser scanning microscope, or a Leica SP8 inverse FALCON microscope (Center for Microscopy and Image Analysis, University of Zurich), in both cases equipped with a Leica APO 63x NA 1.4 oil immersion objective, with images taken at a definition of 1024 x 1024 pixels, and the pinhole diameter adjusted to 1 Airy unit for each emission channel. The quantitative cell image analyses were performed by using ImageJ software and the open-source cell image analysis software CellProfiler™ [6] (Broad Institute, Cambridge, MA).

Immunofluorescent image quantifications.

<u>Quantification of "mean fluorescence intensity"</u>

For all measurement of "mean fluorescence intensity", an outline of each cell was drawn by hand using FIJI (Image J), and the mean fluorescence intensity recorded. For measurements of PI(3,4,5)P_3 and PI(4,5)P_2 intensity in HK2 cells, 10 imaging regions were taken for each condition. For measurement of 488-BSA (binding or internalized) and Cy5-β-lactoglobulin uptake in mPTCs, results were taken from 10 separate imaging regions for each mouse.

<u>Quantification of "puncta per cell"</u>

The pipeline "Speckle counting" in CellProfiler™ was used to the number of PI(3)P or PI(4,5)P_2 puncta surrounding nuclei and to perform per-nuclei aggregate measurements (eg. the number of dots/nucleus), as previously described[5, 6]. Briefly, the images were converted to greyscale using the module "ColorToGray". The "IdentifyPrimaryObjects" module was used to identify nuclei and dots while cells were identified by using the module

“IdentifySecondaryObjects”. “MaskObject” and “RelateObject” modules were used to establish a parent-child relationship between the cells and the dots, identified as masked objects, and to calculate the average of dots per cell.

Quantification of “EEA1/actin overlap” in HK2 cells

The optical airyscan sections just above the section at which stress fibers could be observed were used for the quantification, with the CellProfiler^{TM} pipeline “Cell/particle counting, and scoring the percentage of stained objects” used to score the percentage of EEA1-positive structures colocalizing with F-actin. Briefly, the images were converted to greyscale using the module “ColorToGray”. Then the “ObjectIdentification” module was used to identify the nuclei, the EEA1-positive and the F-actin stained structures/vesicles. The “RelateObject” module was used to establish a parent-child relationship between the stained objects. In this case, a “parent” object (endosomes) is one that touches, overlaps or encloses a “child” object (F-actin structures). When Object1 touches or overlasp with Object2, they are considered to be colocalized and will be assigned as a parent to the corresponding child. The “ClassifyObjects” and “FilterObjects” modules were used to categorize structures that were either colocalizing or not-colocalizing, and to calculate the percentage of colocalizing structures on the total amount of endosomes per image. Approximately 10 images from each sample were analyzed.

Quantification of “EEA1/actin overlap” in mPTCs

Z-stack images acquired by confocal microscope were first deconvolved using Huygens (SVI) software in order to increase object resolution in x, y and z, to improve the signal, and correct the noise. The final z-stack images were loaded into CellProfiler^{TM}, followed by segmentation of images and identification of objects. The process is characterized by three main steps: segmentation of actin and endosomes, identification of overlap objects and tabulation of measurements. The “ObjectIdentification” and “Filter” modules were used to identify the EEA1+ (Endosomes) and the F-actin marked-structures/vesicles. In order to filter

out actin fibers and better visualize the above subcellular EEA1/actin-positive structures, limits in shape (Eccentricity factor below 0.75) and size (Area size in pixel below 80) of the F-actin object were applied. The “RelateObject”, “ClassifyObjects” and “FilterObjects” modules were then used as described above to assign colocalization, “parent” and “child” status, and ultimately the percentage of colocalizing objects. Approximately 14 images from each sample were quantified. The 3D reconstructions of EEA1 positive vesicles and actin were generated in Imaris software (Oxford Instruments) using the full confocal z-stacks (around 45) of each imaging region.

Quantification of “stress fiber score”

The presence of actin fibers in these images was quantified using a custom script in FIJI (ImageJ). Briefly, the script measures directionality of signal without tracing fibers as required for detailed analysis of actin organisation such as that carried out by Rogge et al[7]. It selects the z-slice containing the highest intensity of f-actin labelling and applies Laplacian of Gaussian processing with a sigma value of 2 pixels to suppress noise and detect fibers. It then divides the image into 576 (24x24) tiles of equal area with an overlap factor of 0.2 at each edge, and convolves the tile with each of the 8 Kirsch directional edge detection kernels. The gradient magnitude is the maximum magnitude from any of the 8 resulting images, and its direction is determined by the kernel producing the maximum response. Since we are interested in fiber direction rather than gradient direction, π/2 is added to the kernel direction to give the principal direction of detected edges. The directionality of actin signal is measured as:

$$\text{tile score} = \frac{\max(E) - \min(E)}{\text{sum}(E) - \min(E)} \times 8$$

where *E* is the set of responses in the 8 gradient directions. This scale normalisation gives a measure of relative contribution of the principal direction to the total gradient present in the

tile independent of the magnitude of the gradient. The tile-based approach allows unbiased analysis of small regions of cells to give a set of local measurements representing the degree of directionality of actin signal which can be used to compare the overall level of organisation between conditions.

The script is available on github at https://github.com/gurdon-institute/Stress_Fibre_Scores.

Endocytosis uptake assays. The endocytic capacity of *Ocrl* mouse proximal tubules was examined by measuring Cy5-β-lactoglobulin (L3908, Sigma) uptake as previously described[2]. The endocytic capacity of *Ocrl* mPTCs was examined by measuring albumin uptake using a pulse-chase assay analysed by either immunofluorescence or intracellular fluorescence intensity using a microplate reader[8, 9]. mPTCs were seeded at appropriate densities on either glass coverslips for immunofluorescence experiments or in 96 well plates for plate reader experiments, then treated with 10 μM alpelisib for 16 h. For both types of experiment, cells were washed three times in PBS, then incubated with 0.2 mg/ml Alexa 488-BSA (A13100, Thermo Fisher Scientific) at 4 °C for 1 hour ("pulse"). Following this, cells were rinsed in serum free media and then incubated in standard serum containing media for 20 mins at 37 °C ("chase") to follow the internalization of albumin. For immunofluorescence experiments, cells were washed and fixed (using the 4% formaldehyde fix) at the end of the "pulse", "wash", and "chase" phases of the experiment, labelled for DAPI, and imaged on a laser scanning confocal microscope, then the fluorescence intensity of Alexa 488-BSA in individual cells measured. For measurement of internalized albumin uptake on the plate reader, cells were lysed with RIPA buffer supplemented with protease inhibitors and the fluorescence signal was measured using the microplate reader (Infinite M Plex, Tecan) at an excitation wavelength of 493nm and emission wavelength of 520nm. Protein concentration was determined by bicinchoninic acid (BCA) protein assay kit (Thermo Fischer Scientific). The albumin uptake was normalized to the protein content of the cells in each well.

Western blotting. HK2 cells were lysed in the following buffer: 50 mM Na-HEPES (pH 7.4), 50 mM NaCl, 1 mM EDTA, 10% (v/v) glycerol. 1% (v/v) Triton X-100, Halt-EDTA protease inhibitor tablets for 30 minutes and scraped from the plate. Lysate concentration was determined by BCA protein assay (Pierce) and equal samples loaded and ran on 4-20% mini-PROTEAN polyacrylamide gels (Bio-Rad). Transfer was performed using the iBlot 2 dry blotting system (Life Technologies), blocking was performed in 5% milk-TBST, antibody incubations and washes in 0.5% milk-TBST, and detection was performed using IRDye antigen detection (Goat anti-mouse (926-32210) and anti-rabbit (926-32211) IRDye 800CW) and an Odyssey Sa reader (all LI-COR Biosciences). Densitometry analysis of band intensity was performed from 3-5 biologically independent replicates in Image Studio Lite (software version 5.2.5; LI-COR Biosciences). For mouse work, proteins were extracted from mouse kidney tissues or primary culture cells and western blotting was performed as previously described[3], with the signals revealed using the ChemiDocTM imaging system (Bio-Rad Laboratories) and image intensity was measured in FIJI (ImageJ). The primary antibodies used were as follows: rabbit anti-OCRL ([EP10256], abcam ab 181039) sheep anti-megalin (gift from P. Verroust and R. Kozyraki, INSERM, Paris, France), rabbit anti-Aqp1 (ab2219, Millipore), rabbit anti-PI3 Kinase p110α (4249, Cell Signaling), rabbit anti-PI3 Kinase p110β (3011, Cell Signaling), rabbit anti-PI3 Kinase p110γ (5405, Cell Signaling), rabbit anti-PI3 Kinase p110δ (Cell Signaling) and mouse anti-α-tubulin (T5168, Sigma-Aldrich - used in mouse experiments or Abcam ab7291 - used in HK2 experiments).

Data analysis and Statistics. The quantitative data were expressed as means ± standard error of the mean (SEM). Differences between experimental groups were evaluated using one-way analysis of variance (ANOVA) followed by Bonferroni test or Kruskal Wallis followed by Dunn's multiple comparison test, or unpaired or paired two tailed Student's t-test, when appropriate, and as indicated in the figure legends. The sample size of each experimental group is described in the figure legends. GraphPad Prism software was used for all statistical analyses. Statistical significance was set at a $P<0.05$.

References

1. Daste F, Walrant A, Holst MR*, et al.* Control of actin polymerization via the coincidence of phosphoinositides and high membrane curvature. *The Journal of cell biology* 2017; **216:** 3745-3765.

2. Festa BP, Berquez M, Gassama A*, et al.* OCRL Deficiency Impairs Endolysosomal Function in a Humanized Mouse Model for Lowe Syndrome and Dent Disease. *Human molecular genetics* 2018.

3. Festa BP, Chen Z, Berquez M*, et al.* Impaired autophagy bridges lysosomal storage disease and epithelial dysfunction in the kidney. *Nature communications* 2018; **9:** 161.

4. Hammond GR, Schiavo G, Irvine RF. Immunocytochemical techniques reveal multiple, distinct cellular pools of PtdIns4P and PtdIns(4,5)P(2). *The Biochemical journal* 2009; **422:** 23-35.

5. De Leo MG, Staiano L, Vicinanza M*, et al.* Autophagosome-lysosome fusion triggers a lysosomal response mediated by TLR9 and controlled by OCRL. *Nature cell biology* 2016; **18:** 839-850.

6. Carpenter AE, Jones TR, Lamprecht MR*, et al.* CellProfiler: image analysis software for identifying and quantifying cell phenotypes. *Genome biology* 2006; **7:** R100.

7. Rogge H, Artelt N, Endlich N*, et al.* Automated segmentation and quantification of actin stress fibres undergoing experimentally induced changes. *Journal of microscopy* 2017; **268:** 129-140.

8. Terryn S, Jouret F, Vandenabeele F*, et al.* A primary culture of mouse proximal tubular cells, established on collagen-coated membranes. *American journal of physiology Renal physiology* 2007; **293:** F476-485.

9. Wong DW, Yiu WH, Wu HJ*, et al.* Downregulation of renal tubular Wnt/beta-catenin signaling by Dickkopf-3 induces tubular cell death in proteinuric nephropathy. *Cell death & disease* 2016; **7:** e2155.

Supplementary Video 1. Imaris 3D rendering of EEA1/actin overlap in *Ocrl*$^{Y/+}$ mPTCs treated with DMSO.

Representative 3D surface rendering of a confocal stack of *Ocrl*$^{Y/+}$ mPTCs treated with DMSO for 16 hours then fixed with the 4% formaldehyde fix and immunolabelled for EEA1 (purple), actin [phalloidin] (yellow) and DAPI (blue), illustrating little actin/endosomal association. Scale bar = 5μm.

Supplementary Video 2. Imaris 3D rendering of EEA1/actin overlap in *Ocrl*$^{Y/-}$ mPTCs treated with DMSO.

Representative 3D surface rendering of a confocal stack of *Ocrl*$^{Y/-}$ mPTCs treated with DMSO for 16 hours then fixed with the 4% formaldehyde fix and immunolabelled for EEA1 (purple), actin [phalloidin] (yellow) and DAPI (blue), illustrating punctate actin associating near endosomal structures. Scale bar = 5μm.

Supplementary Video 3. Imaris 3D rendering of EEA1/actin overlap in *Ocrl*$^{Y/-}$ mPTCs treated with alpelisib.

Representative 3D surface rendering of a confocal stack of *Ocrl*$^{Y/-}$ mPTCs treated with 10 μM of alpelisib for 16 hours then fixed with the 4% formaldehyde fix and immunolabelled for EEA1 (purple), actin [phalloidin] (yellow) and DAPI (blue), illustrating rescue of actin punctate strucures around endosomes. Scale bar = 5μm.

Supplementary Table 1

	Baseline (BL)				Day 14				Day 28				Day 42 (D42)			
	Ocrl$^{Y/+}$		*Ocrl*$^{Y/-}$		*Ocrl*$^{Y/+}$		*Ocrl*$^{Y/-}$		*Ocrl*$^{Y/+}$		*Ocrl*$^{Y/-}$		*Ocrl*$^{Y/+}$		*Ocrl*$^{Y/-}$	
	Vehicle n=8	Alpelisib n=8	Vehicle n=8	Alpelisib n=6	Vehicle n=8	Alpelisib n=8	Vehicle n=8	Alpelisib n=6	Vehicle n=8	Alpelisib n=8	Vehicle n=8	Alpelisib n=6	Vehicle n=8	Alpelisib n=8	Vehicle n=8	Alpelisib n=6
Body weight (gr.)	22.4 ± 0.5	22.8 ± 0.3	23.4 ± 0.4	22.0 ± 1.1	25.2 ± 0.7	24.2 ± 0.3	26.8 ± 0.5	**22.8 ± 1.9**[a]	26.5 ± 0.8	25.9 ± 0.4	27.8 ± 0.5	24.5 ± 1.7	28.5 ± 0.7	27.4 ± 0.3	29.8 ± 0.5	**25.9 ± 1.9**[a]
Urinary volume (µL/15h)	1386 ±152	1190 ±156	1644 ±236	1998 ±182	1895 ± 222	1638 ± 260	1784 ± 122	1383 ± 247	1214 ± 161	1180 ± 171	1270 ± 138	1100 ±121	1279 ± 57	1635 ± 217	1650 ± 218	1633 ± 239
Diuresis (µL/gr. B.W./min)	0.07 ± 0.01	0.06 ± 0.01	0.08 ± 0.01	0.10 ± 0.01	0.08 ± 0.01	0.08 ± 0.01	0.07 ±0.005	0.06 ± 0.01	0.05 ± 0.01	0.05 ± 0.01	0.05 ± 0.01	0.05 ±0.003	0.05 ± 0.01	0.07 ± 0.01	0.06 ± 0.01	0.07 ± 0.01
U Creatinine (mg/dL)	22.6 ±1.6	22.2 ± 2.0	19.1 ± 2.2	17.5 ± 1.2	20.3 ± 2.1	19.6 ± 2.6	19.8 ± 0.9	18.3 ± 2.2	25.7 ± 3.1	20.4 ± 1.4	21.6 ± 2.1	16.1 ± 2.3	21.9 ± 1.4	19.8 ± 1.3	23.5 ± 2.2	16.7 ± 2.6
U glucose (mg/15h)	0.33 ± 0.08	0.48 ± 0.15	0.42 ± 0.16	0.53 ± 0.14	0.42 ± 0.08	**77.40 ± 30.99**[a]	0.65 ± 0.39	**31.18 ± 14.31**[a]	0.23 ± 0.03	**36.21 ± 16.19**[a]	0.18 ± 0.03	6.95 ± 3,74	0.18 ± 0.02	**53.28 ± 15.35**[b]	0.27 ± 0.05	**30.56 ±14.69**[a]
CC16 (µg/15h)	0	0	1.30 ± 0.27	1.91 ± 0.21	-	-	0.81 ± 0.09	0.85 ± 0.10	-	-	0.95 ± 0.24	0.55 ± 0.11	-	-	1.31 ± 0.18	1.248 ± 0.20
U albumin (µg/15h)	3.1 ± 0.8	2.5 ± 0.6	14.6 ± 2.7	20.8 ± 2.4	2.7 ± 0.8	3.7 ± 1.4	13.4 ± 1.6	17.0 ± 4.0	2.4 ± 0.5	7.1 ± 3.1	7.0 ± 0.9	8.1 ± 1.6	2.0 ± 0.6	1.3 ± 0.5	9.6 ± 1.8	13.1 ± 2.4
U phosphate (mg/15h)	1.5 ± 0.2	1.4 ± 0.2	1.5 ± 0.2	1.8 ± 0.2	1.3 ± 0.1	1.3 ± 0.1	1.2 ± 0.1	1.0 ± 0.2	1.2 ± 0.1	1.2 ± 0.2	1.1 ± 0.2	0.8 ± 0.2	1.1 ± 0.1	1.6 ±0.2	1.2 ± 0.1	1.3 ± 0.3
Glycemia (mmol/L)	-	-	-	-	-	-	-	-	-	-	-	-	8.7 ± 0.3	**7.2 ±0.4**[a]	8.5 ± 0.3	**6.4 ± 0.7**[a]
BUN (mg/dL)	-	-	-	-	-	-	-	-	-	-	-	-	25.8 ± 2.0	20.3 ± 1.9	25.4 ± 0.7	**19.2 ± 1.5**[a]

Supplementary Table 1. Body weight, urine and blood parameters over time in *Ocrl* mice treated with alpelisib or vehicle.

Body weight, urine and blood parameters in *Ocrl* mice treated as indicated at the time points indicated. Key: U, Urine; CC16, Clara cell protein 16; BUN, Blood urea nitrogen; Plotted data represent mean ± SEM. Indicated comparisons were performed by two-tailed unpaired Student's t-test applied between vehicle and alpelisib for each genotype for each timepoint. ^{a} $p< 0.05$, ^{b} $p< 0.01$, versus *Ocrl* $^{Y/+}$ vehicle or *Ocrl* $^{Y/-}$ vehicle as appropriate.

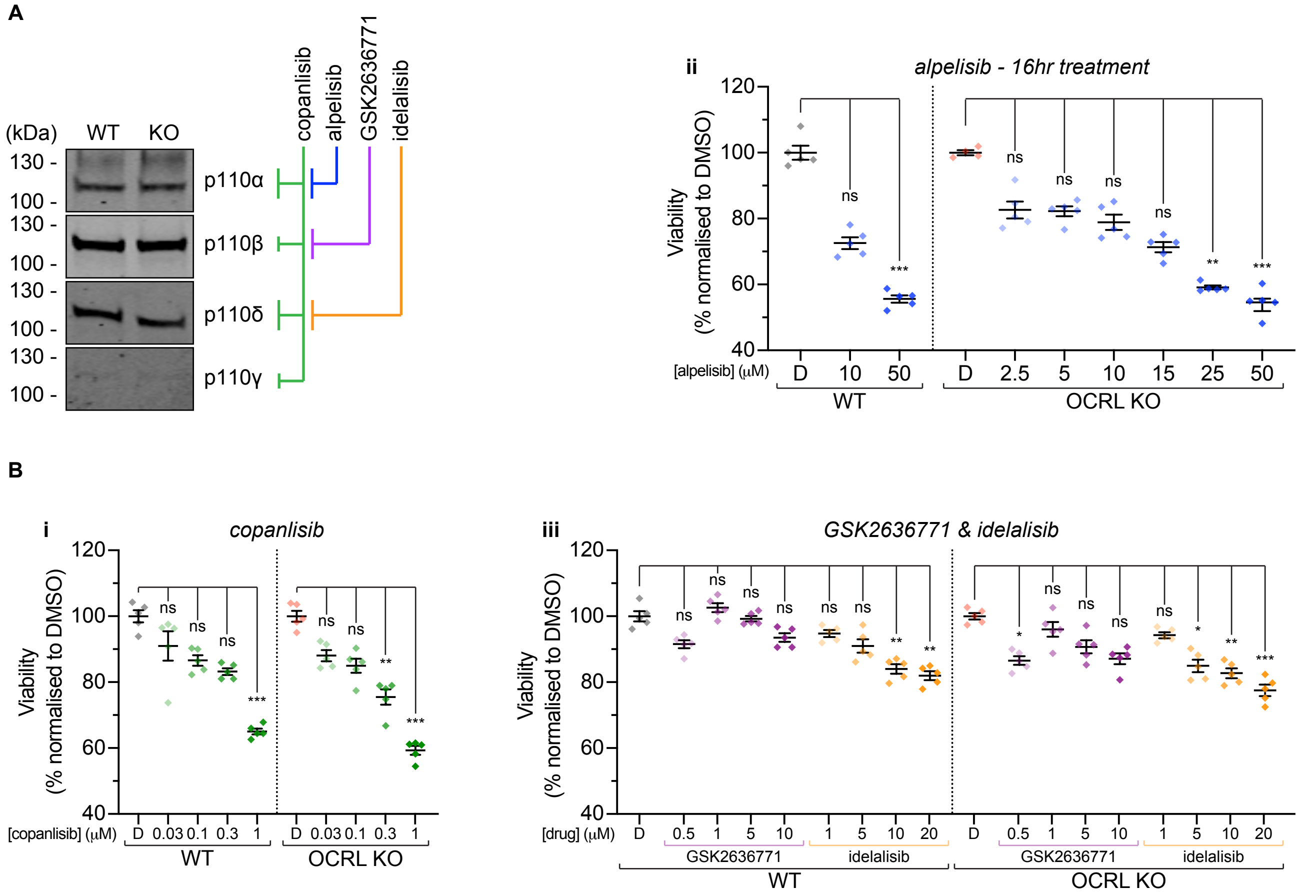

Supplementary Figure 1. p110 isoform expression and drug dose response cytotoxicity assays in HK2 cells.

(A) Western blots illustrating the expression pattern of each of the four p110 class I PI3K catalytic isoforms (α, β, γ and δ) in HK2 cells. With the exception of PI3Kγ the different isoforms are well expressed in both WT and KO cell lines. The blot for α-tubulin providing a loading control is shown in Figure 1B. The schematic illustrates the p110 isoform specificity of each the PI3K inhibitors used in this study. **(B)** Dose response MTT assays to assess cytotoxicity of (i) copanlisib, (ii) alpelisib, and (iii) GSK2636771 and idelalisib at the indicated doses for 16 hours in HK2 WT control and OCRL KO cells. In all cases viability has been normalized to the mean of the corresponding DMSO control. N=4-5 wells, lines indicate mean ± SEM, data points indicate individual wells. Statistical significance was assessed by Kruskal-Wallis (K-W) ANOVA with Dunn's multiple comparison test to compare each dose with the appropriate DMSO control. (i) [copanlisib], overall Kruskal-Wallis test: P<0.0001 (***), multiple comparisons: WT DMSO vs 0.03 μM copanlisib P>0.999 (ns), vs 0.1 μM P=0.4728 (ns), vs 0.3 μM P=0.1138 (ns), vs 1 μM P=0.0006 (***), KO DMSO vs 0.03 μM P>0.9463 (ns), vs 0.1 μM P=0.2826 (ns), vs 0.3 μM P=0.0067 (**), vs 1 μM P<0.0001 (***). (ii) [16hr alpelisib], overall Kruskal-Wallis test: P<0.0001 (***), multiple comparisons: WT DMSO vs 10 μM alpelisib. P=0.1543 (ns), vs 50 μM P=0.0003 (***), KO DMSO vs 2.5 μM P>0.9999 (ns), vs 5 μM P>0.9999 (ns), vs 10 μM P>0.9999 (ns), vs 15 μM P<0.0945 (ns),vs 25 μM P=0.0037 (**), vs 50 μM P=0.0003 (ns). (iii) [16hr GSK2636771 and idelalisib], overall Kruskal-Wallis test: P<0.0001 (***), multiple comparisons: WT DMSO vs 0.5, 1, 5 and 10 μM GSK2636771, KO DMSO vs 1 μM GSK26336771 all P>0.9999 (ns), KO DMSO vs 0.5 μM GSK26336771, P=0. 0366 (*), KO DMSO vs 5 μM GSK2636771, P=0. 5067 (ns) KO DMSO vs 10 μM GSK26336771, P=0. 0672 (ns), WT DMSO vs 1 μM idelalisib P>0.9999 (ns), vs 5 μM idelalisib P>0.7758 (ns), vs 10 μM idelalisib P>0.0082 (**), vs 20 μM idelalisib P>0.0020 (**), KO DMSO vs 1 μM idelalisib P>0.9999 (ns), vs 5 μM idelalisib P>0.0117 (*), vs 10 μM idelalisib P>0.0022 (**), vs 20 μM idelalisib P>0.0002 (***).

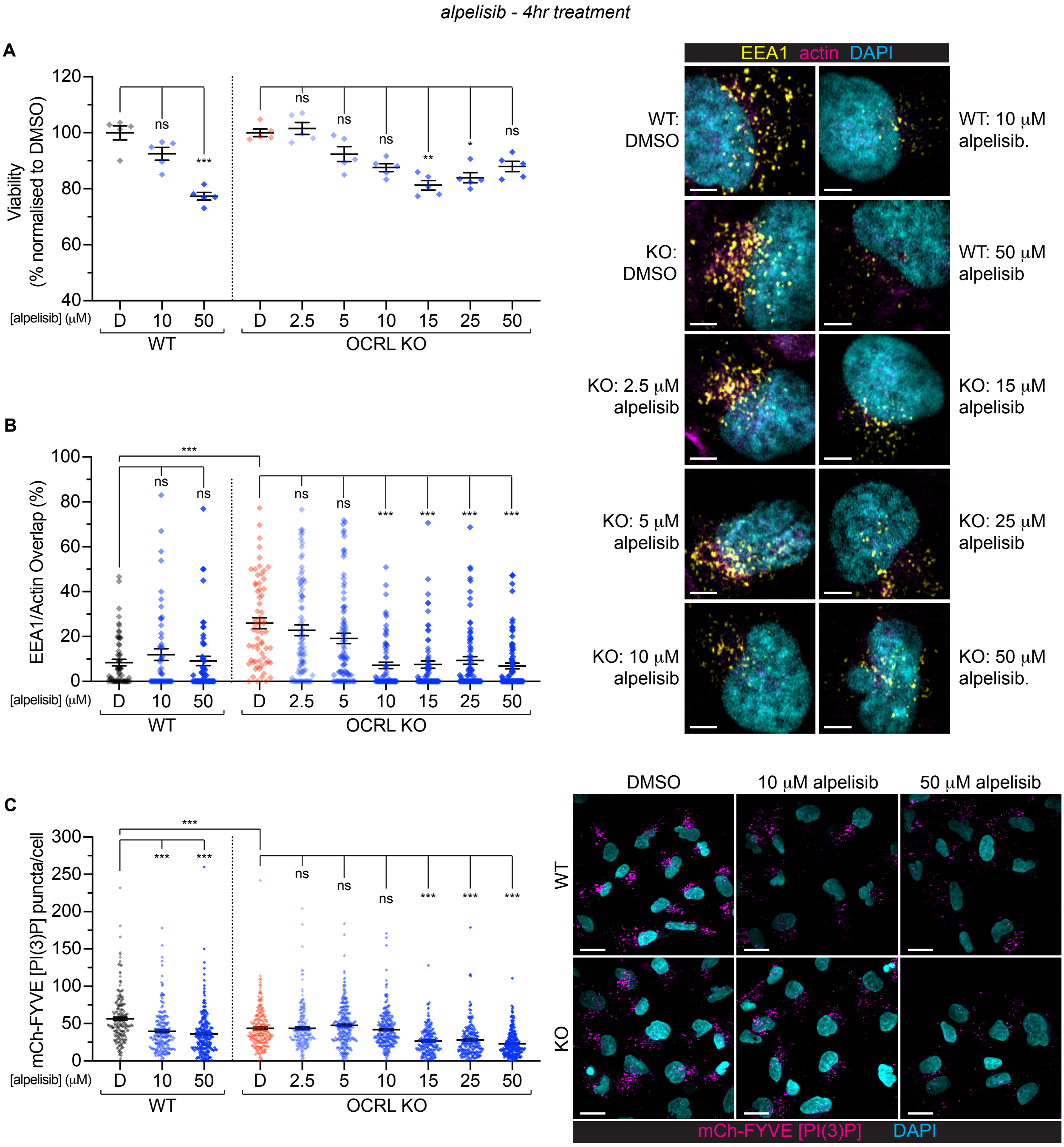

Supplementary Figure 2. PI3K inhibitor alpelisib restores actin-endosomal overlap and reduces PI(3)P levels in a dose responsive manner four hours after drug treatment in HK2 cells.

(A) Dose response MTT assay to assess cytotoxicity of alpelisib (4 hour treatment) at the indicated doses in HK2 WT control and OCRL KO cells. Viability has been normalized to the mean of the DMSO WT control. N=5 wells, lines indicate mean ± SEM, data points indicate individual wells. Statistical significance was assessed by K-W ANOVA with Dunn's multiple comparison test to compare each dose with the appropriate DMSO control; overall: $P<0.0001$ (***), multiple comparisons: WT DMSO vs 10 µM alpelisib $P>0.999$ (ns), vs 50 µM $P=0.0005$ (***), KO DMSO vs 2.5 µM $P>0.9999$ (ns), vs 5 µM $P>0.9999$ (ns), vs 10 µM $P=0.3128$ (ns), vs 15 µM $P<0.0095$ (**),vs 25 µM $P=0.0374$ (*), vs 50 µM $P=0.3785$ (ns). **(B)** Representative Airyscan confocal micrographs (fixed using the 4% formaldehyde fix and immunolabelled for EEA1, yellow, actin [phalloidin], magenta and DAPI, cyan, scale bars = 5 µm) and quantification of WT or KO HK2 cells treated with DMSO or alpelisib at the indicated doses for 4 hours, demonstrating dose responsive rescue of the actin-endosomal overlap. In all cases lines indicated mean ± SEM, points indicate individual cells.

(N = 62, 54, 55, 64, 81, 82, 71, 69, 79, 81 cells for WT control, WT 10 μM, WT 50 μM, KO DMSO, KO 2.5, 5, 10, 15, 25 and 50 μM alpelisib respectively. Statistical significance assessed by Kruskal-Wallis test: overall P<0.001 (***), multiple comparisons; WT DMSO vs. KO DMSO, KO DMSO vs KO 10, 15, 25 and 50 μM alpelisib all P<0.001 (***), KO DMSO vs KO 2.5 μM alpelisib P=0.6170 (ns), KO DMSO vs KO 5 μM alpelisib P=0.0576 (ns), WT DMSO vs both WT 10 and 50 μM alpelisib P>0.9999 (ns). (C) Representative confocal micrographs of WT or KO HK2 cells treated with either DMSO, 10 or 50 μM alpelisib for 4 hours then fixed with the golgi fix and labelled using the mCh-2xFYVE PI(3)P probe (magenta) and DAPI (cyan), with quantification of the number of PI(3)P positive puncta detected in cells treated with a range of alpelisib concentrations, as indicated, showing PI(3)P positive puncta are reduced in a dose responsive fashion. N = 280, 254, 210, 287, 324, 239, 340, 250, 240 & 215 cells for WT control, WT 10 μM, WT 50 μM, KO DMSO, KO 2.5, 5, 10, 15, 25 and 50 μM alpelisib respectively. Statistical significance was assessed by K-W ANOVA: overall P<0.001 (***), multiple comparisons; WT DMSO vs. KO DMSO, P=0.0003, WT DMSO vs WT 10 and 50 μM alpelisib plus KO DMSO vs KO 15, 25 and 50 μM alpelisib all P<0.001 (***), KO DMSO vs KO 2.5, 5 and 10 μM alpelisib all P>0.9999 (ns).

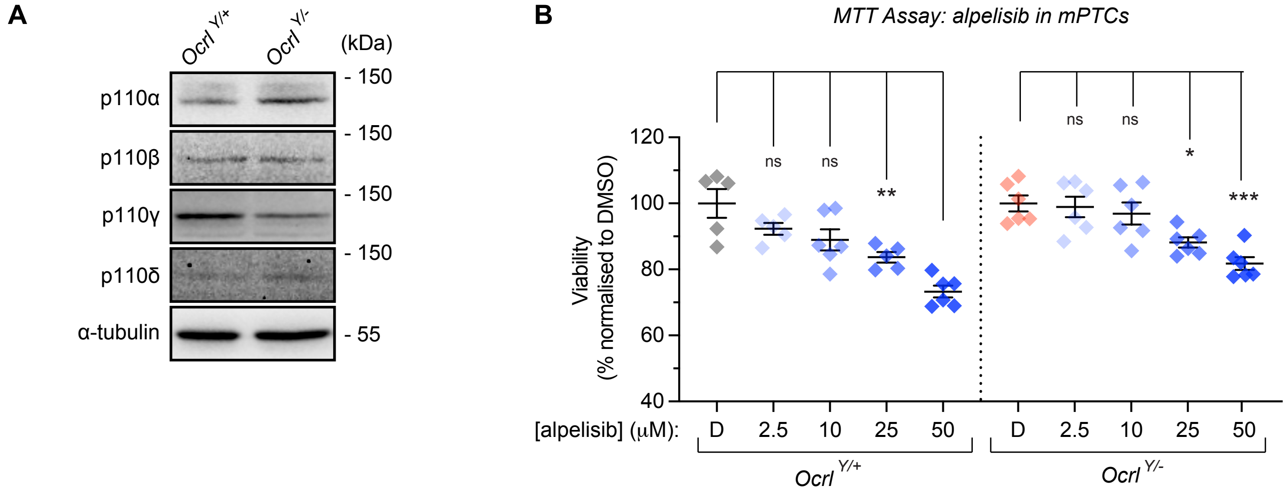

Supplementary Figure 3. p110 isoform expression and alpelisib toxicity in *Ocrl* mPTCs.

(A) Western blotting of p110α, p110β, p110γ, p110δ levels in mPTCs derived from *Ocrl* mice. **(B)** MTT assay of *Ocrl* mPTCs treated either with DMSO or with the indicated concentration of alpelisib overnight. In all cases viability has been normalized to the mean of the corresponding DMSO control. N=5 wells, lines indicate mean ± SEM, data points indicate individual wells. Statistical significance was assessed by one–way ANOVA (overall P<0.0001 [***]) followed by Bonferroni's post hoc tests to compare each dosage with its respective DMSO control, all comparisons indicated as P>0.05 (ns), P<0.05 (*), P<0.01 (**) and P<0.001 (***) relative to *Ocrl*$^{Y/+}$ or *Ocrl*$^{Y/-}$ mPTCs.

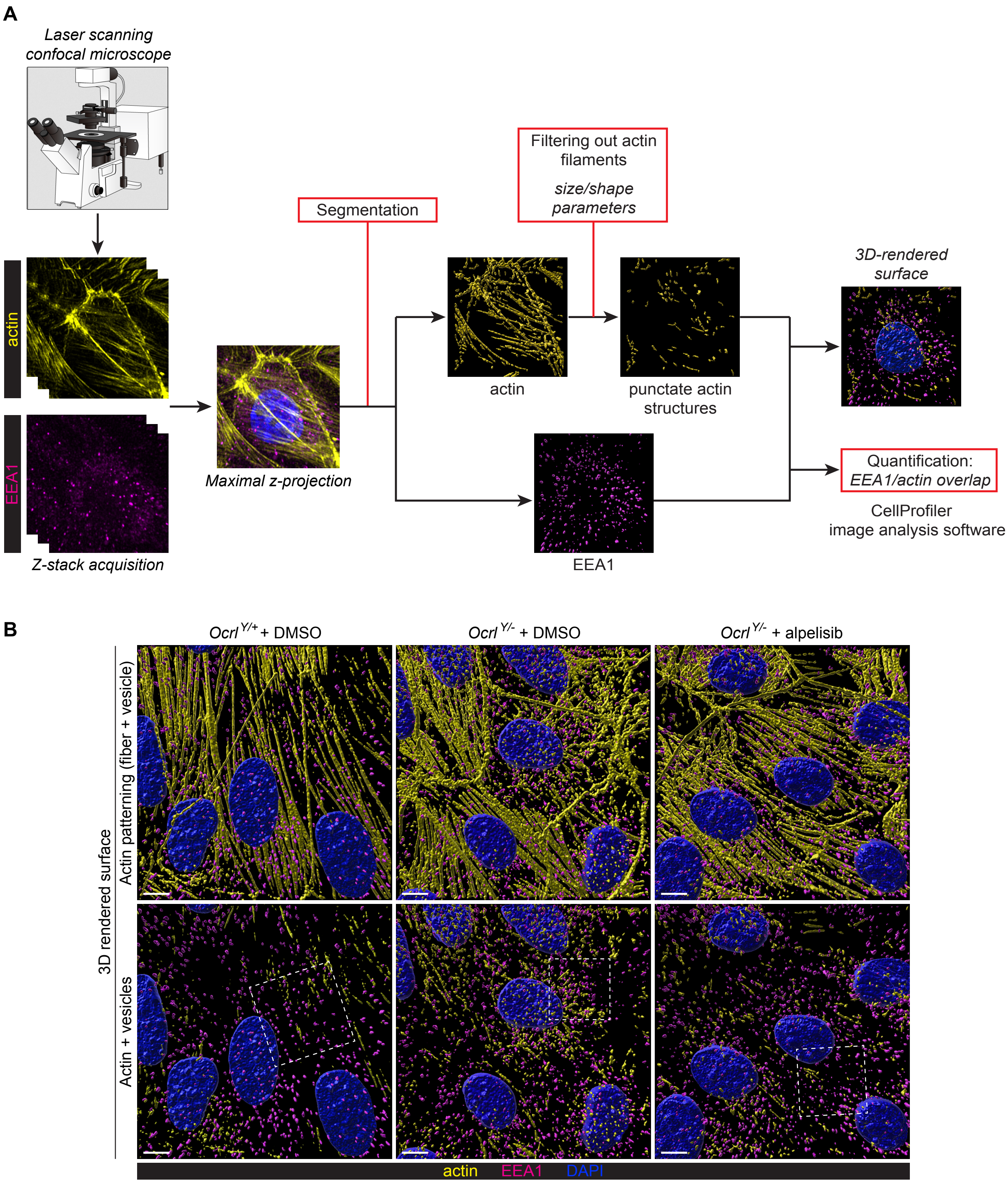

Supplementary Figure 4. Workflow of EEA1/actin overlap analysis in *Ocrl* mPTCs.

(A) Workflow of the strategy used to generate 3D renderings for segmentation to allow quantification of the number of EEA1/actin-positive structures in mPTCs. **(B)** Representative 3D surface renderings of *Ocrl* mPTCs treated with DMSO or 10 μM of alpelisib for 16 hours then fixed with the 4% formaldehyde fix and immunolabelled for EEA1 (purple), actin (phalloidin, yellow) and DAPI (blue), illustrating the initial 3D surface renderings (top panel), followed by the output of the size/shape parameter filtering step (bottom panel) used for quantification of actin/vesicle overlap. The boxes indicate the regions shown in the high magnification views in Figure 4B. Scale bars = 5 μm.

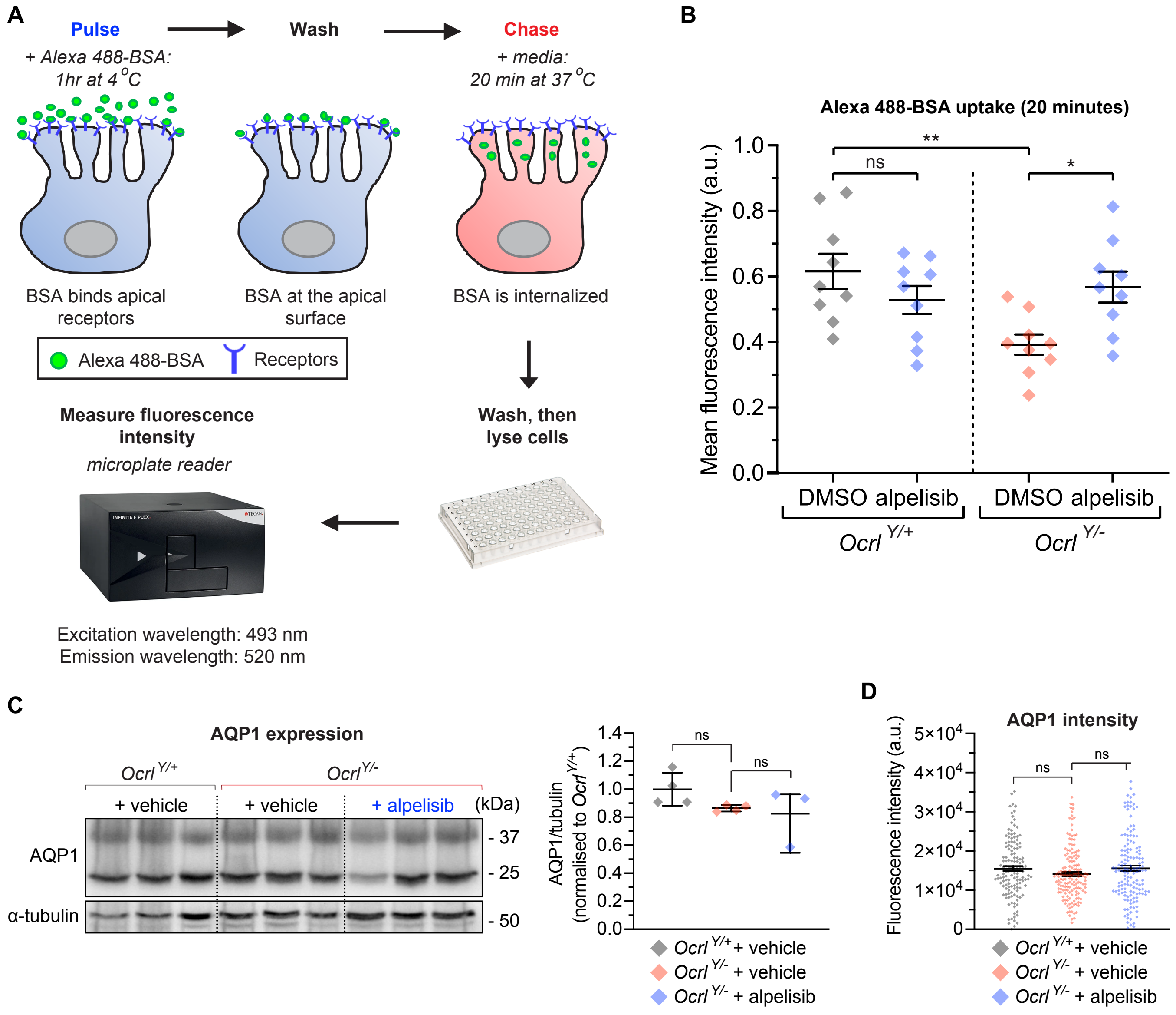

Supplementary Figure 5. Alpelisib restores albumin uptake in *Ocrl*$^{Y/-}$ mPTCs. Aqp1 expression is unaffected.

(A) Workflow of the strategy used to assess Alexa 488-BSA uptake via microplate reader. Cells were loaded with Alexa 488-BSA (0.2 mg ml^{-1}) for 1h at 37°C, with albumin uptake followed by incubating the cells at 37°C for 20 min in cell medium before lysis and measuring fluorescence intensity on the plate reader. **(B)** Quantification of Alexa 488-BSA uptake fluorescence intensity, illustrating that alpelisib rescues BSA uptake. N=9 wells pooled from four mouse kidneys per condition; each data point represents the mean fluorescence intensity in an individual well. Significance was tested by K-W ANOVA with Dunn's multiple comparisons test: overall P<0.01 (*), multiple comparisons: *Ocrl*$^{Y/+}$ DMSO vs *Ocrl*$^{Y/-}$ DMSO, P=0.006 (**), *Ocrl*$^{Y/-}$ DMSO vs *Ocrl*$^{Y/-}$ alpelisib P=0.03 (*), *Ocrl*$^{Y/+}$ DMSO vs *Ocrl*$^{Y/+}$ alpelisib P>0.99 (ns). **(C)** Western blotting and densitometry analysis of AQP1 levels in whole kidney lysates from *Ocrl* mice. α-tubulin was used as a loading control. AQP1 levels are constant across the conditions. In the quantification of the densitometry analysis each dot represents one mouse (N=4 *Ocrl*$^{Y/+}$ + vehicle and *Ocrl*$^{Y/-}$ + vehicle and N=3 *Ocrl*$^{Y/-}$ + alpelisib mice), lines indicate mean ± SEM. Significance was assessed by two-tailed unpaired Student's t-tests: *Ocrl*$^{Y/+}$ + vehicle vs *Ocrl*$^{Y/-}$ + vehicle, P=0.0654 (ns), *Ocrl*$^{Y/-}$ + vehicle vs *Ocrl*$^{Y/-}$ + alpelisib, P=0.719 (ns). **(D)** Quantification of AQP1 intensity in confocal images immunolabelled for megalin, AQP1 and DAPI (representative example images shown in Fig. 6G), illustrating no change in levels across the treatments. N = 136, 155 and 144 tubules respectively for *Ocrl*$^{Y/+}$+ vehicle, *Ocrl*$^{Y/-}$ + vehicle and *Ocrl*$^{Y/-}$ + alpelisib for 3 mice per treatment group. Each dot represents fluorescence intensity normalized by tubule area; plotted data indicates the mean ± SEM. Significance was assessed by K-W followed by Dunn's multiple comparison test; P=0.16 (ns), multiple comparisons, *Ocrl*$^{Y/+}$ + vehicle vs *Ocrl*$^{Y/-}$ + vehicle, P=0.20 (ns), *Ocrl*$^{Y/-}$ + vehicle vs *Ocrl*$^{Y/-}$ + alpelisib, P=0.48 (ns).
